# Characterization of cellular senescence patterns predicts the prognosis and therapeutic response of hepatocellular carcinoma

**DOI:** 10.3389/fmolb.2022.1100285

**Published:** 2022-12-16

**Authors:** Yuqin Tang, Chengbin Guo, Chuanliang Chen, Yongqiang Zhang

**Affiliations:** ^1^ Clinical Bioinformatics Experimental Center, Henan Provincial People’s Hospital, Zhengzhou University, Zhengzhou, China; ^2^ Faculty of Medicine, Macau University of Science and Technology, Macau, China; ^3^ Guangzhou Women and Children’s Medical Center, Guangzhou Medical University, Guangzhou, China

**Keywords:** cellular senescence, immune microenvironment, therapeutic response, prognosis, hepatocellular carcinoma

## Abstract

**Background:** Hepatocellular carcinoma (HCC) is a prevalent malignancy with a high mortality rate. Cellular senescence, an irreversible state of cell cycle arrest, plays a paradoxical role in cancer progression. Here, we aimed to identify Hepatocellular carcinoma subtypes by cellular senescence-related genes (CSGs) and to construct a cellular senescence-related gene subtype predictor as well as a novel prognostic scoring system, which was expected to predict clinical outcomes and therapeutic response of Hepatocellular carcinoma.

**Methods:** RNA-seq data and clinical information of Hepatocellular carcinoma patients were derived from The Cancer Genome Atlas (TCGA) and International Cancer Genome Consortium (ICGC). The “multi-split” selection was used to screen the robust prognostic cellular senescence-related genes. Unsupervised clustering was performed to identify CSGs-related subtypes and a discriminant model was obtained through multiple statistical approaches. A CSGs-based prognostic model-CSGscore, was constructed by LASSO-Cox regression and stepwise regression. Immunophenoscore (IPS) and Tumor Immune Dysfunction and Exclusion (TIDE) were utilized to evaluate the immunotherapy response. Tumor stemness indices mRNAsi and mDNAsi were used to analyze the relationship between CSGscore and stemness.

**Results:** 238 robust prognostic differentially expressed cellular senescence-related genes (DECSGs) were used to categorize all 336 hepatocellular carcinoma patients of the TCGA-LIHC cohort into two groups with different survival. Two hub genes, TOP2A and KIF11 were confirmed as key indicators and were used to form a precise and concise cellular senescence-related gene subtype predictor. Five genes (PSRC1, SOCS2, TMEM45A, CCT5, and STC2) were selected from the TCGA training dataset to construct the prognostic CSGscore signature, which could precisely predict the prognosis of hepatocellular carcinoma patients both in the training and validation datasets. Multivariate analysis verified it as an independent prognostic factor. Besides, CSGscore was also a valuable predictor of therapeutic responses in hepatocellular carcinoma. More downstream analysis revealed the signature genes were significantly associated with stemness and tumor progression.

**Conclusion:** Two subtypes with divergent outcomes were identified by prognostic cellular senescence-related genes and based on that, a subtype indicator was established. Moreover, a prognostic CSGscore system was constructed to predict the survival outcomes and sensitivity of therapeutic responses in hepatocellular carcinoma, providing novel insight into hepatocellular carcinoma biomarkers investigation and design of tailored treatments depending on the molecular characteristics of individual patients.

## 1 Introduction

Hepatocellular carcinoma (HCC) is the most prevalent type of liver cancer, the fourth leading cause of death among all cancers, and its incidence is rising rapidly in recent years (Couri and Pillai, 2019; Yang et al., 2019). In the past decades, tremendous progress has been made in epidemiology, risk factors, and molecular and genetic profiles of HCC, contributing to the evolution of prevention, surveillance, early diagnosis, and treatment (Huang et al., 2020; Petrick et al., 2020). Surgical intervention, including surgical resection and liver transplantation, is the best choice of treatment for patients with early liver cancer and the only way to enable patients to achieve long-term survival and even cure (Fan, 2012; Fonseca and Cha, 2014). However, surgical intervention is restricted to a small proportion of HCC patients with extremely specific clinical characteristics (Forner et al., 2010). Since sorafenib, a molecularly targeted drug for advanced HCC, was approved in 2007, the research and development of cancer-targeted drugs have been a hot topic worldwide (Cheng et al., 2009). Moreover, tumor immunotherapy is in the ascendant, encouraging us to start a new era of cancer treatment, mainly including programmed cell death 1 (PD-1) and cytotoxic T lymphocyte-associated antigen 4 (CTLA-4) checkpoint inhibitors (Sangro et al., 2013; Chang Lee and Tebbutt, 2019). As far as we know, the heterogeneity of the HCC tumor microenvironment (TME) leads to different therapeutic effects of targeted drugs and immunocheckpoint inhibitors (ICIs) on HCC patients. Therefore, the molecular profiling and subtype identification of HCC patients could contribute to the efficacy of personalized treatment and prognostic prediction.

Cell senescence is a process in which cells stop dividing or lose their proliferative capacity, can be induced by a couple of stresses such as the activation of oncogenes and DNA damage caused by conventional chemotherapies/radiotherapies (Fei and El-Deiry, 2003; Gewirtz et al., 2008; Ewald et al., 2010; Petrova et al., 2016). Therefore, cell senescence was once considered a defense mechanism against cancer because of its ability to maintain a stable cell cycle arrest (Campisi, 2013). Tumor-suppression characteristic of cell senescence has paved the way for a novel idea that enhances the senescence in tumor cells for cancer patients (Gertler et al., 2004; Collado et al., 2005; Calcinotto and Alimonti, 2017). However, a growing number of studies pointed out that cellular senescence caused genomic perturbations and played a paradoxical role in tumorigenesis, being both tumor-promoting and tumor-suppressive (Perez-Mancera et al., 2014; Ruhland et al., 2016; Ou et al., 2021). In terms of tumor-promoting, senescent cancer cells remain metabolically active and secrete multiple types of factors, such as cytokines, chemokines, and proteases, known as the senescence-associated secretory phenotype (SASP), which can induce the proliferation of neighboring non-senescent cancer cells (Kuilman et al., 2010; Jackson et al., 2012).

**FIGURE 1:**
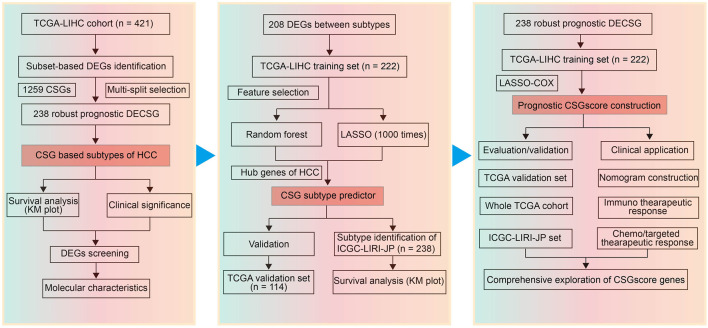
Flowchart of this study.

Triggered by the p53 tumor suppressor, cellular senescence has been recognized as a suppressive factor of HCC by inducing the cell-autonomous program of cell-cycle arrest or apoptosis (Kang et al., 2011; Lujambio et al., 2013). In line with the finding that SASP can promote tumorigenesis by disturbing the tumor immune microenvironment (TIME), several studies reported that cellular senescence was capable of promoting HCC progression (Yoshimoto et al., 2013; Ohtani, 2014; Huang et al., 2021). In this study, we attempted to reveal the influence of cellular senescence-related genes (CSGs) on the prognosis of HCC, which would provide novel biomarkers for CSGs-related prediction for the prognosis and therapeutic response of HCC. With tumor-specific CSGs obtained from the intersection between a senescence-related gene set and the differentially expressed genes (DEGs) in the TCGA-LIHC cohort, we used an unsupervised clustering method to identify two CSGs-related subtypes with divergent clinical outcomes in HCC. Next, LASSO (Least absolute shrinkage and selection operator) Cox regression and random forest were performed, and then TOP2A and KIF11 were found as key indicators for CSGs-related subtype classification. Based on the two subtypes, a five-gene scoring system called “CSGscore” was established to predict the survival status in HCC. We confirmed the reliability and robustness of CSGscore for the prognostic prediction with internal and external datasets. Moreover, we observed the potential of CSGscore in predicting HCC patients’ response to immunotherapy or chemo-/targeted therapy. The flowchart of this study was shown in Figure 1.

## 2 Materials and methods

### 2.1 Data acquisition and preprocessing

We downloaded the gene expression files of 370 HCC and 50 adjacent normal tissues from the TCGA database (https://portal.gdc.cancer.gov/) using the “TCGAbiolinks” package (Colaprico et al., 2016). The corresponding clinicopathologic characteristics and their survival information including overall survival (OS), disease-free survival (DFS), progression-free survival (PFS), and disease-specific survival (DSS) were also gathered. The one-class logistic regression (OCLR)-based mRNAsi and mDNAsi for evaluating the differentiation degree from bulk sequencing were obtained as previously described (Malta et al., 2018). For survival-related analysis, 336 of 370 patients with the survival time of >1 month were included (Cai et al., 2020; Guo et al., 2022), which were divided into the training and validation datasets with the ratio of 2:1 (222 vs*.* 114). The transcriptome profiles and survival data of 238 HCC patients from the International Cancer Genome Consortium (ICGC) database (ICGC-LIRI-JP) were acquired to serve as the external validation set. All data were normalized and processed as previously reported (Guo et al., 2022; Tang et al., 2022). 1,259 cellular senescence-related genes (CSGs) including 525 positive CSGs and 734 negative CSGs were retrieved from a recently published paper (Wang et al., 2022). All the patient data used for model construction in the current study were summarized in Supplementary Table S1.

### 2.2 Identification of prognostic differentially expressed CSGs (DECSGs) and unsupervised clustering

Differentially expressed genes (DEGs) between HCC and adjacent normal tissues were screened by using the “limma” package (Ritchie et al., 2015), with the criteria cutoff of adj.P.Val<0.01 and | logFC|>1. To balance the samples between the tumor and normal groups, we utilized the subset-based method as we previously described (Guo et al., 2021). Generally, five subsets were randomly generated from the tumor group with the sample size of 74, yielding a ratio of 1.48:1 for tumor and normal. The common DEGs in the five subset-based analyses were obtained by plotting a Venn diagram, which were further intersected with 1,259 CSGs (Wang et al., 2022) to acquire the DECSGs in HCC. Subsequently, Kaplan-Meier survival analysis and log-rank test were used to identify the prognostic DECSGs for OS. To enhance the stability, we adopted the “multi-split” approach as we reported with some modifications (Tang et al., 2020; Guo et al., 2022; Zhang et al., 2022). Next, the prognostic DECSGs were utilized to perform unsupervised clustering for the patients with complete survival information, and the R package “ConsensusClusterPlus” was used to explore novel CSG patterns of HCC (Wilkerson and Hayes, 2010; Wang et al., 2021; Guo et al., 2022).

### 2.3 DEGs between HCC subtypes and function enrichment

The package “limma” was utilized to screen the DEGs between two CSG subtypes of HCC, followed by the enrichment analysis of biological functions and pathways. The “clusterProfiler” R package was applied to conduct the Gene Ontology (GO) enrichment and the most significant terms were visualized by using the “ggplot2” package. Meanwhile, the online DAVID tool (https://david.ncifcrf.gov/tools.jsp) was used to perform the Kyoto Encyclopedia of Genes and Genomes (KEGG) pathway analysis, and a network showing the most significant enriched pathways was drawn by the package “ggraph.”

### 2.4 Gene set variation analysis (GSVA) and gene set enrichment analysis (GSEA)

The Gene set variation analysis process was completed to calculate the enrichment scores of 50 hallmark gene sets of the molecular signature database (Liberzon et al., 2015) for the TCGA-LIHC cohort by the “GSVA” package (Hanzelmann et al., 2013). Differential analysis was then performed by the “limma” package to reveal the relative activities of these hallmark pathways in the two CSG subtypes, which was determined by the criterion of *p* < 0.01 combined with absolute *t* > 2.5 (Lambrechts et al., 2018; Zhang et al., 2022). GSEA was further employed to verify the GSVA results using the GSEA software with default settings. The cutoff of FDR <0.25 was considered significant as recommended (Zhang et al., 2022).

### 2.5 Building a CSG subtype predictor in HCC

In this section, all 336 samples from the TCGA-LIHC cohort were randomly classified into the training set (*n* = 222) and the validation set (*n* = 114) at a ratio of 2:1, and the ICGC-LIRI-JP dataset (*n* = 238) was selected as an external validation set. In the training set, two feature selection algorithms suitable for high dimensionality were applied to screen the most subtype-relevant features: LASSO regression and the Random Forest. The expression of the top 208 DEGs between two CSG subtypes with the cutoff of |logFC| > 1.5 and adj.P.Val<0.01 was used as the input variable and the CSG subtype status was used as the binary outcome (0 or 1). For the LASSO selection operator, the “multi-split” strategy was adopted to minimize the effect of arbitrary choice in the random sample split process (Xu et al., 2017; Luo et al., 2020). We subsampled 75% of the training dataset 1,000 times and only those repeatedly occurred more than 700 times were selected. Moreover, random forest was carried out for variable elimination with the OOB error as the minimization criterion. The intersecting genes were further narrowed down by comparing them with the hub genes in HCC that were reported before (Tang et al., 2020; Zhang et al., 2021). Afterward, two overlapping critical genes were used to fit a binary prediction model with multivariate logistic regression analysis (Xu et al., 2017; Luo et al., 2020; Wang et al., 2021). The discriminative performance of the CSG subtype predictor was investigated by the receiver operating characteristic (ROC) curves and the areas under the ROC curve (AUCs) in the training and validation datasets. The optimal cutoff value derived from the training set according to the maximum Youden’s index was applied to the training and validation datasets to generate the predicted outcomes and confusion matrixes. Finally, the CSG subtypes for the external dataset ICGC-LIHC-JP were predicted in a similar way and the prognostic value of the CSG subtype was further validated.

### 2.6 Prognostic CSGs scoring system (CSGscore) establishment

We examined the utility of the CSGs for the construction of a prognostic CSGs scoring system with the aforementioned prognostic DECSGs. Based on the TCGA training dataset, all the 238 robust prognostic DECSGs were subjected to LASSO-COX and stepwise regression algorithms to generate the best subset of prognostic genes, which were subsequently used to build the prognostic CSGs signature: CSGscore = Σ(coef (β)*EXP_β_), where β indicates each selected prognostic DECSG. Next, the CSGscores for the HCC patients in the training, validation, and external dataset were computed, and all the patients were categorized into the high- and low-risk groups depending on the median CSGscores in the training set. Kaplan-Meier survival curves and log-rank tests were used to compare the divergent survival outcomes in both groups. Time-dependent receiver operating characteristic (tROC) curves were drawn to evaluate the predictive power of CSGscore. Besides, stratified survival analysis was used to explore its additional prognostic value in subgroups divided by clinicopathologic variables.

Univariate and multivariate analyses were conducted to verify the independent prognostic capacity of CSGscore. Based on the results of Univariate analysis, a CSGscore-integrated nomogram was established with the “rms” package. Calibration curves were plotted to estimate the consistency between predicted probabilities of 3- and 5-year survival and actual ones. The decision curve analysis (DCA) curves at 1-, 3-, and 5- years were depicted to compare the net benefits of the nomogram and that of the pathologic stage or tumor burden status. Kaplan-Meier analysis was further applied to test the clinical relevance of the nomogram for OS, DFS, PFS, and DSS.

### 2.7 Therapeutic sensitivities prediction by CSGscore

Based on the whole TCGA-LIHC cohort, the potential role of CSGscore in predicting the immunotherapeutic efficacy of HCC was preliminarily assessed by comparing the gene expression of 50 immune checkpoint genes (ICGs) between distinct CSGscore groups. Immunophenoscore (IPS) representing the tumor immunogenicity of each sample was acquired from The Cancer Immunome Atlas (TCIA) (https://tcia.at/home), and a lower IPS stands for worse sensitivities to immunotherapy (Charoentong et al., 2017). The tumor immune dysfunction and exclusion (TIDE) score, which was designed to predict the influences on survival and immunotherapeutic responses based on two mechanisms of immune evasion: T-cell exclusion and T-cell dysfunction (Jiang et al., 2018), was computed with the expression profiles of the whole TCGA-LIHC cohort and the ICGC-LIRI-JP dataset. Wilcoxon test was used to compare the TIDE scores between the high- and low- CSGscore groups, and Chi-square test was used to analyze the ratio of responders or non-responders in both groups. Meanwhile, the “pRRophetic” package was used to calculate the semi-inhibitory concentration (IC_50_) values of 138 chemo/targeted drugs (Geeleher et al., 2014).

### 2.8 Correlation of CSGscore with immune infiltration

For the immune infiltration landscape, we utilized the CIBERSORT algorithm (Newman et al., 2015) to estimate the relative percentage of 22 tumor immune cell types based on the whole TCGA-LIHC cohort. The tumor immune cell infiltration for the high- and low-risk groups were computed and presented with a heatmap. Spearman correlation analysis was used to investigate the correlation of immune cell infiltration and CSGscore as well as the expression of CSGscore genes, followed by the visualization of a correlation heatmap.

### 2.9 Comprehensive exploration of the CSGscore genes

The innovative OCLR machine-learning algorithm-derived stemness indices including mRNAsi and mDNAsi have correlated with multiple clinical observations in malignant tumors such as poor survival, tumor metastasis, and therapeutic resistance (Malta et al., 2018; Wang et al., 2021; Huang et al., 2022). Thus, we used the whole TCGA-LIHC cohort to compare the mRNAsi and mDNAsi in the two CSGscore risk groups by Wilcoxon test. Spearman correlation analysis was then performed to validate the relationship between each CSGscore gene and mRNAsi or mDNAsi. The differential expression patterns of these CSGscore genes were further verified by paired HCC and non-cancerous tissue samples (*n* = 50). Finally, the correlation of CSGscore genes’ expression and clinicopathologic parameters including tumor grade, pathologic stage, tumor burden status, and CSG subtype was also investigated *via* boxplots.

### 2.10 Statistical analysis

Kaplan-Meier and log-rank tests were used to compare the survival outcomes for OS, DFS, PFS, and DSS in different groups. Wilcoxon test or Kruskal-Wallis test was used for the comparisons of continuous variables in two or three groups. Correlation coefficients and statistical significance were calculated by Spearman correlation analysis. Pearson Chi-square test was utilized to examine the distribution differences of categorical variables. The R package “glmnet” and “randomForest” were used to perform the LASSO and random forest algorithms for dimensionality reduction. The “pROC” package was used to assess the predictive accuracies for the CSG subtype predictor and the “timeROC” package was used to determine the prognostic reliability of the CSGs scoring system. All data visualization was completed in RStudio (Version 1.1.383, https://www.rstudio.com/).

## 3 Results

### 3.1 Identification of CSG subtypes of HCC

**FIGURE 2:**
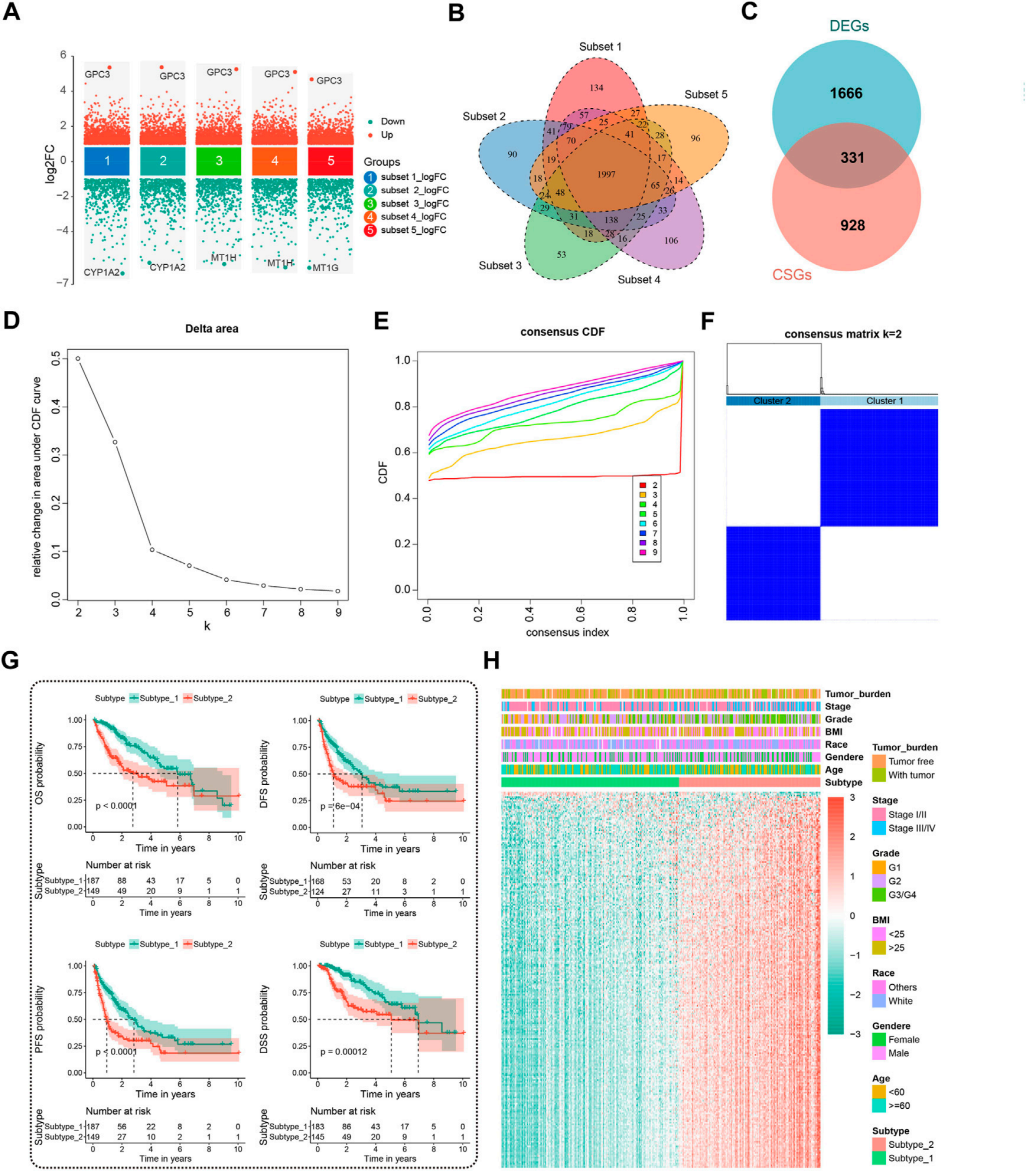
Identification of prognostic DECSGs and consensus clustering. **(A)** Screening of the DEGs in HCC with the subset-based approach. **(B)** Venn diagram showing the 1997 DEGs shared by the five subset-based DEGs. **(C)** Venn diagram showing the 331 DECSGs in HCC. **(D–F)** Two subtypes of HCC were recognized with 238 robust prognostic CSGs using unsupervised consensus clustering. **(G)** Kaplan-Meier survival plots of two CSG subtypes for OS, DFS, PFS, and DSS. **(H)** The 238 gene profiles of two CSG subtypes with clinical traits. DECSGs, differentially expressed cellular senescence genes; DEGs, differentially expressed genes; OS, overall survival; DFS, disease-free survival; PFS, progression-free survival; DSS, disease-specific survival.

Applying the “limma” package with the screening criteria of |logFC| > 1 and adj.P.Val <0.01, we extracted 1997 overlapping DEGs among five randomly subsampled subsets from TCGA-LIHC dataset using the subset-based approach (Figures 2A,B). Next, we intersected the previously reported 1,259 cellular senescence genes (CSGs) with these 1997 DEGs to obtain 331 DEGs associated with CS (DECSGs) (Figure 2C). Currently, a growing body of evidence has linked CS with cancer progression and metastasis, which in turn affects the prognosis (Wang et al., 2022). Therefore, we screened the robust DECSGs significantly correlated to the prognosis of HCC patients by the “multi-split” method, and 238 DECSGs were observed to be associated with prognosis in more than 990 of 1,000 subsamples (Supplementary Table S2), and by those genes, two subtypes of HCC were recognized by unsupervised clustering analysis (Figures 2D–F, Supplementary Figure S1). Furthermore, by utilizing Kaplan-Meier analysis, we found that patients in the two subtypes had distinct clinical outcomes, and patients in subtype 2 had a significantly shorter survival time than the patients in subtype 1 (Figure 2G). We also exhibited the gene profiles of the 238 DECSGs in the two HCC subtypes along with clinical traits (Figure 2H).

### 3.2 Clinical significance and biological functions of the CSG subtypes of HCC

**FIGURE 3:**
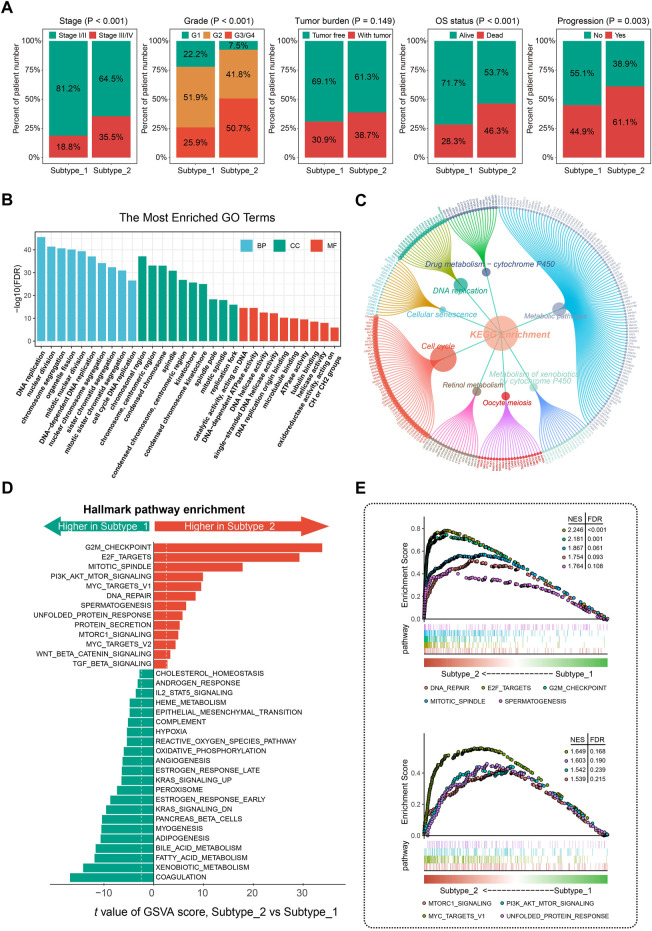
Clinical significance and molecular characteristics of CSG patterns of HCC. **(A)** Proportions of subgroups divided by tumor stage, grade, tumor burden, OS status, and progression status in different CSG subtypes. **(B,C)** GO **(B)** and KEGG **(C)** enrichment analysis of the DEGs between CSG subtypes. **(D,E)** Significant hallmark pathways enriched by GSVA **(D)** and GSEA **(E)** between CSG subtypes. GO, gene ontology; KEGG, Kyoto Encyclopedia of Genes and Genomes; GSVA, gene set variation analysis; GSEA, gene set enrichment analysis.

Differential analysis of clinicopathologic characteristics between the two HCC subtypes from the TCGA-LIHC cohort was performed. Notably, Chi-square test results showed significantly differential sample distribution for pathologic stage, tumor grade, OS status, and progression status of HCC concerning the defined CSG subtype. (Figure 3A). More patients with advanced stage (35.5% vs. 18.8%), high grade (50.7% vs. 25.9%), and poor progression status (61.1% vs. 44.9%) were clustered into subtype 2 than subtype 1, corresponding to the poorer prognosis of subtype 2.

GO enrichment analysis revealed that the most significant terms enriched by the DEGs between the two subtypes were the biological process (BP) of DNA replication, cellular component (CC) of chromosomal region, and molecular function (MF) of catalytic activity acting on DNA (Figure 3B). For the KEGG enrichment analysis, DEGs between the two CSG subtypes mostly participated in the pathways of metabolic pathways and cell cycle (Figure 3C). GSVA regarding hallmark gene sets was performed in the two HCC subtypes. As Figure 3D shows, the CSG subtype 2 was significantly associated with several cell cycle-related pathways such as G2M checkpoint, E2F targets, and mitotic spindle pathway while those from subtype 1 were associated with immune- and metabolism-related pathways such as coagulation, xenobiotic metabolism, and fatty acid metabolism pathway. Meanwhile, GSEA confirmed the differences in hallmark pathways enriched in the two HCC subtypes. Similar to the results of GSVA analysis, we observed that multiple cell cycle- and cancer-related pathways were more frequently enriched in subtype 2 (Figure 3E).

### 3.3 Construction of CSGs-related subtype predictor

**FIGURE 4:**
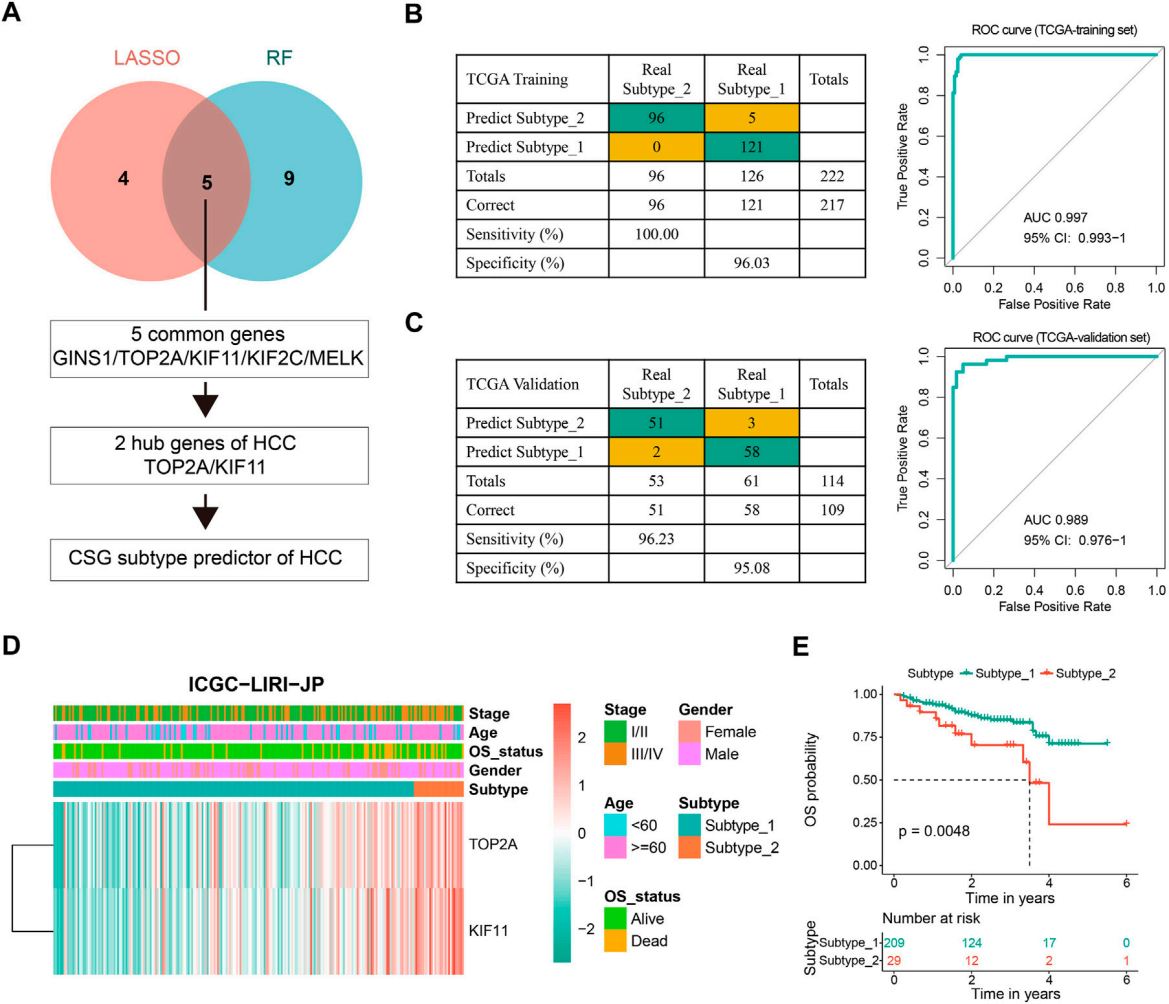
Construction and evaluation of the CSG subtype predictor of HCC. **(A)** Workflow for building the CSG subtype predictor. **(B,C)** Confusion matrixes and ROC curves of the CSG subtype predictor for the binary classification in the TCGA training **(B)** and validation **(C)** datasets. **(D)** Unsupervised hierarchical clustering of the two selected hub genes in the CSG subtype predictor for the ICGC-LIRI-JP dataset. **(E)** Kaplan-Meier survival plots showing the significantly different OS between the two CSG subtypes.

Preliminary work inspired us to investigate potential biomarkers with indicative roles in the classification of CSGs-related HCC subtypes to achieve a precise and concise model for CSG subtype categorization of HCC. We first picked out the top 208 of 784 DEGs between the two HCC subtypes with the cutoff of |logFC| > 1.5 (Supplementary Table S3). Next, we conducted LASSO regression and random forest with the 208 DEGs and screened out nine and 14 variables, respectively. Five genes (GINS1, TOP2A, KIF11, KIF2C, and MELK) were found in common by both algorithms. Interestingly, TOP2A and KIF11 were widely recognized as hub genes of HCC, which were also presented to be strongly associated with the CSGs-related subtype classification. Figure 4A outlined the screening process of these two critical hub genes (TOP2A and KIF11), which were further used to construct the CSGs-related subtype predictor. Confusion matrixes showed that the expression of TOP2A and KIF11 in HCC patients could precisely predict the CSGs-related subtype in the TCGA training cohort (totally 217 of 222 samples were correctly predicted) and the TCGA validation cohort (totally 109 of 114 samples were correctly predicted) and ROC curves also demonstrated the reliability of the two-gene subtype predictor (Figures 4B,C). Moreover, the confusion matrix and ROC curve for the whole TCGA-LIHC cohort were shown in Supplementary Figure S2. Additionally, we performed CSG subtype prediction with the subtype predictor for the ICGC-LIRI-JP dataset and 238 patients were successfully classified into two groups with distinct OS status (Figures 4D,E).

### 3.4 Construction and validation of the CSGscore model

**FIGURE 5:**
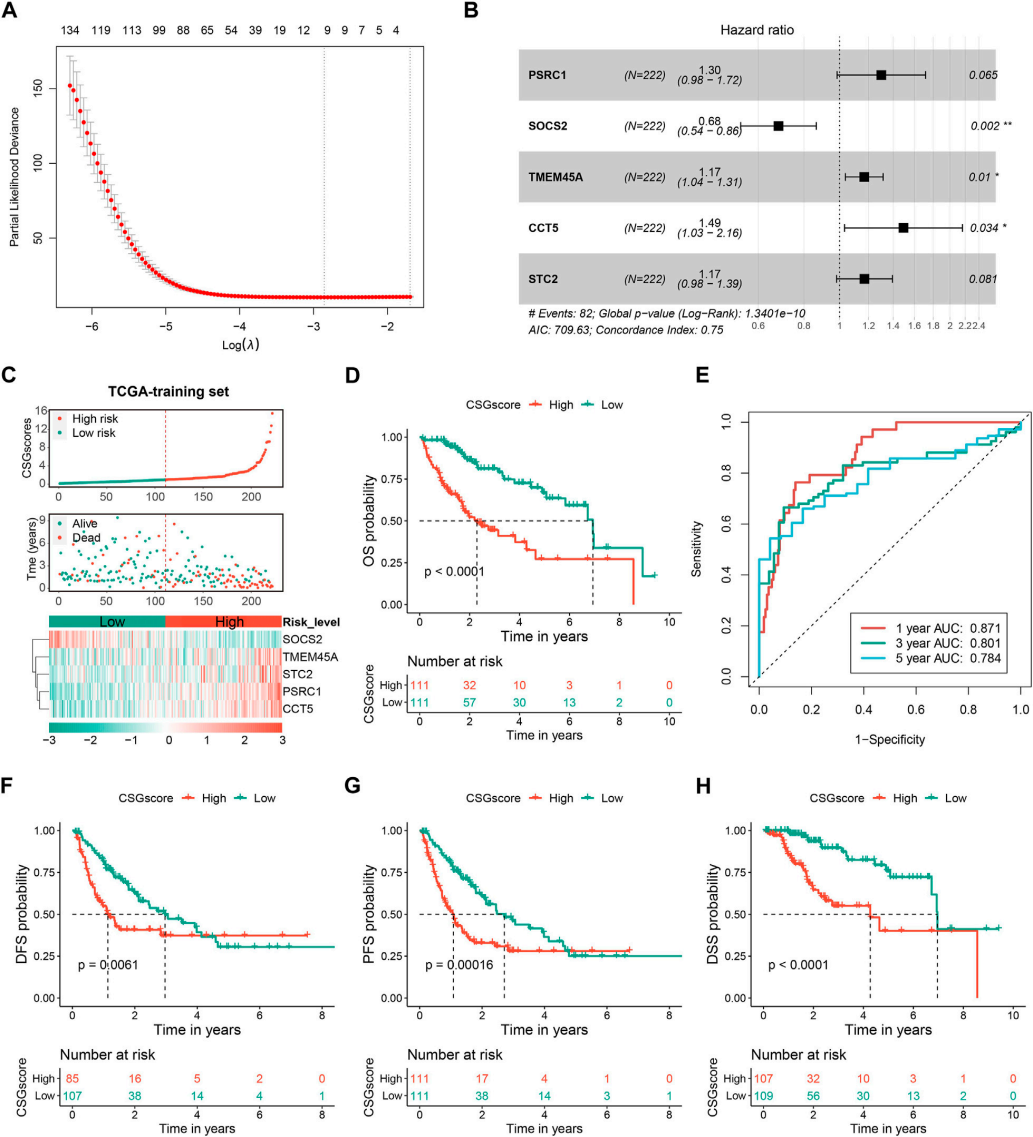
Construction and evaluation of the CSGs scoring system (CSGscore) of HCC based on the TCGA training dataset**. (A)** LASSO-COX was applied to identify 10 potential genes, followed by the stepwise method to select the final five of them for the construction of the CSGs scoring system. **(B)** Forest plot showing the coefficients and significant levels of the five CSGscore genes in the model. **(C)** Patients in the TCGA training set were classified into the high- and low-risk groups according to the median value of CSGscores, and **(C)** shows the distribution of their CSGscores, survival status, and the expression profile of the five CSGscore genes. **(D,E)** The Kaplan-Meier survival plots **(D)** and tROC curves **(E)** for the OS of CSGscore in the training dataset. **(F–H)** The Kaplan-Meier survival plots of CSGscore for the DFS, PFS, and DSS of HCC.

To evaluate the capability of CSGs in the prognosis prediction of HCC, the abovementioned 238 robust prognostic DECSGs were applied as the input variables for the LASSO-COX regression analysis to construct a prognostic model, which resulted in 10 potential genes for the next incorporation into the stepwise regression (Figure 5A). The final model consisting of PSRC1, SOCS2, TMEM45A, CCT5, and STC2 generated a minimal AIC value (AIC = 709.63). Therefore, the final prognostic CSGscore risk model was constructed with the five prognostic CSGs, whose coefficients and significance levels in the model were shown in Figure 5B. To evaluate the prognostic value of the CSGscore model for HCC, all patients in the TCGA training set were classified into high- and low-risk groups according to the median value of CSGscore (0.9824545), and the CSGscore distribution, survival status, and expression profile of HCC patients were exhibited Figure 5C. As shown in Figure 5D, Kaplan-Meier analysis showed that high-risk patients had exceedingly lower OS rates relative to low-risk patients in the TCGA training cohort. The time-dependent receiver operating characteristic curve showed the area under the ROC curve (AUC) of 0.871, 0.801, and 0.784 in 1-year, 3-year, and 5-year OS, respectively, suggesting the high accuracy of CSGscore for OS prediction (Figure 5E). Moreover, we also found the high-risk group had a worse prognosis for DFS, PFS, and DSS (Figures 5F–H). Notably, stratified analysis indicates the CSGscore signature could recognize different risk status significantly in all subgroups divided by clinicopathologic parameters, which denotes its good potential and clinical application to exert additional prognostic value to existing risk factors (Supplementary Figure S3).

**FIGURE 6:**
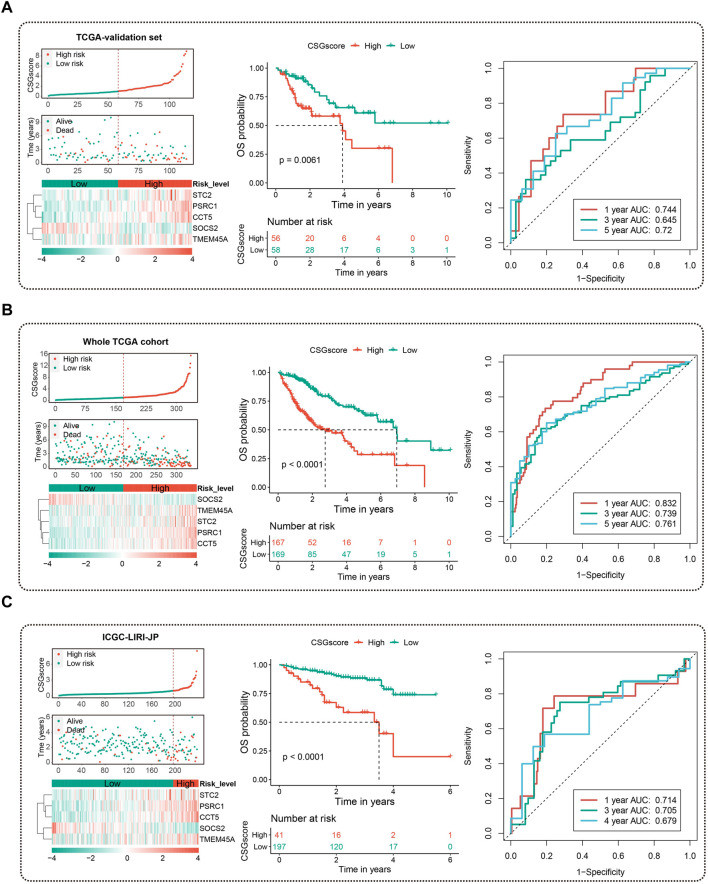
Validation of the prognostic value of CSGscore in HCC. **(A–C)** The left panels indicate the distribution of CSGscore, survival status, and the expression of the five CSGscore genes in the TCGA validation set **(A)**, the whole TCGA cohort **(B)**, and the ICGC-LIRI-JP dataset **(C)**. The middle and right panels indicate the Kaplan-Meier survival plots (the middle panels) and the tROC (the right panels) of CSGscore for the OS of the TCGA validation set **(A)**, the whole TCGA cohort **(B)**, and the ICGC-LIRI-JP dataset **(C)**.

To validate the stability and robustness of CSGscore for OS prediction, we adopted the TCGA-validation dataset and the whole TCGA cohort as internal validation sets and chose the ICGC-LIRI-JP dataset as an external set. Using the same cutoff value that was derived from the training set (0.9824545), all patients in each validation set were categorized into high- and low-risk groups. As Figure 6 shown, the CSGscore was consistent in predicting the prognosis of HCC in all of them, and CSGscore-defined high-risk patients had a significantly worse prognosis than the low-risk group.

**FIGURE 7:**
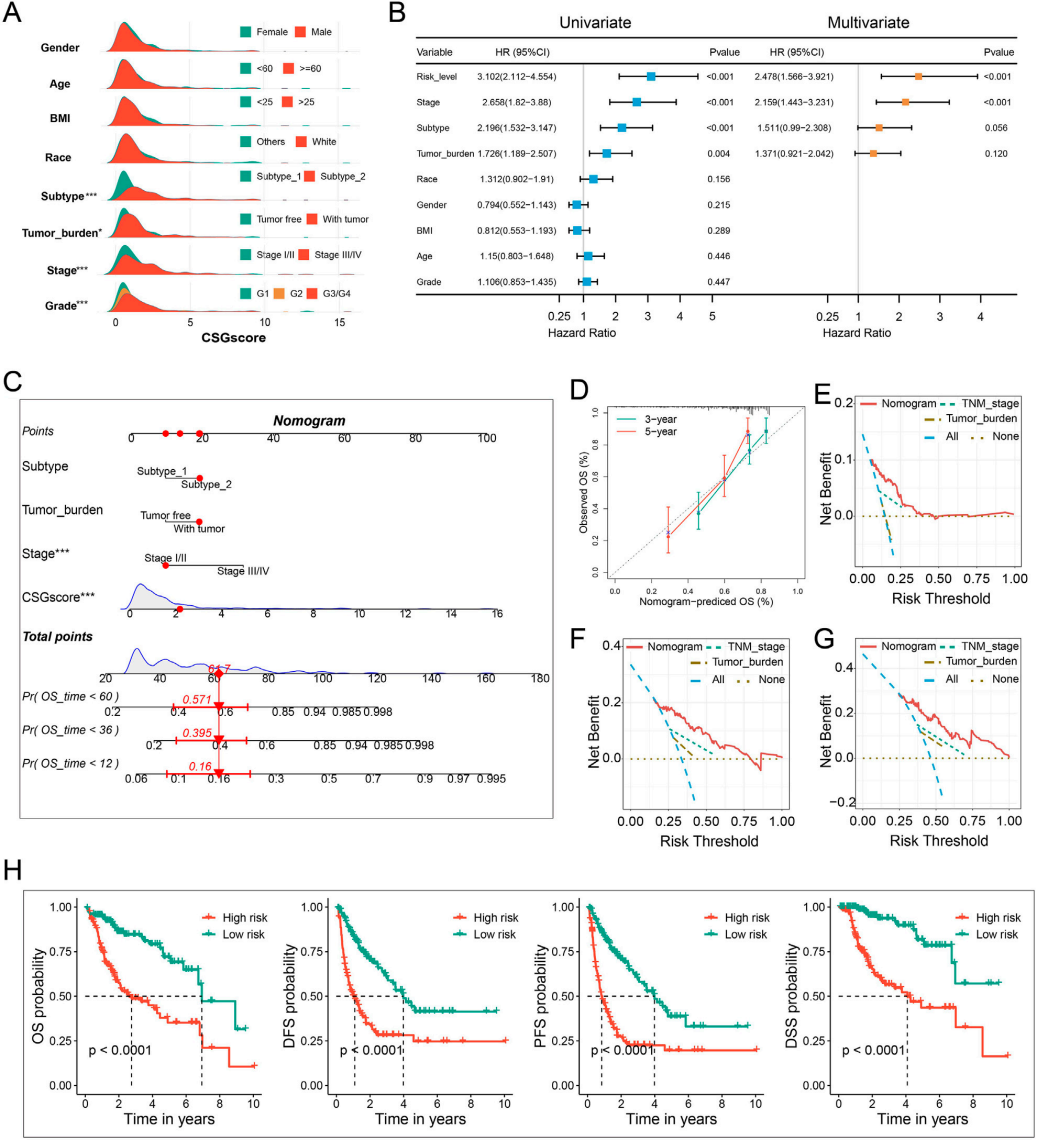
Integration of clinicopathologic characteristics and CSGscore to construct the nomogram. **(A)** Comparison of CSGscore distribution based on clinicopathologic parameters using the whole TCGA cohort. **(B)** Univariate and multivariate analysis of CSGscore and other clinicopathologic traits based on the whole TCGA cohort. **(C)** The CSGscore-integrated nomogram. **(D)** Calibration curves of the nomogram. **(E–G)** Decision curve analysis (DCA) plot of the nomogram for 1-year **(E)**, 3-year **(F)**, and 5-year **(G)** OS prediction. **(H)** Kaplan-Meier curves of the nomogram for the OS, DFS, PFS, and DSS of HCC. *p < 0.05, **p < 0.01, ***p < 0.001, and ns indicates no significance.

The correlation between CSGscore and clinicopathologic features was subsequently explored. As presented in Figure 7A and Supplementary Figure S4, the CSG subtype, tumor burden, stage, and tumor grade were significantly related to CSGscore, while other clinical characteristics showed no significant association with CSGscore.

### 3.5 CSGscore-integrated nomogram establishment

The accurate preoperative prediction of OS might help surgeons to make better clinical decisions and comprehensive nomograms can be used for the combined diagnosis or disease status prediction with multiple indicators. In this situation, accuracy was of the essence, so we were prompted to construct a novel nomogram combing the CSGscore model and multiple clinicopathologic traits to constitute a quantitative tool for predicting the clinical outcomes of HCC patients. While univariate analysis identified the CSGscore classifier, stage, CSG subtype, and tumor burden were prognostic variables, in multivariate analysis, only CSGscore and tumor stage were proved to be independent prognostic indicators for HCC (Figure 7B). Afterward, by integrating the prognostic factors including CSGscore, stage, tumor burden, and CSG subtypes, we established an integrative nomogram to predict the 1-, 3-, and 5-year OS of HCC patients (Figure 7C). Well-fitted calibration curves demonstrated that the nomogram had excellent consistency between the predicted and observed 3- and 5-year OS (Figure 7D). Moreover, The 1-, 3-, and 5-year DCA curves showed that the CSGscore-integrated nomogram could yield better net benefits compared with other indices for the vast majority of threshold probabilities (Figures 7E–G). Importantly, Kaplan-Meier analyses of OS, DFS, PFS, and DSS for low- and high-risk HCC patients classified by the median value of the nomogram score suggested that the CSGscore-integrated nomogram was a utilizable tool in evaluating the prognosis of HCC patients (Figure 7H).

### 3.6 CSGscore correlates with tumor immune infiltration and therapeutic responses

**FIGURE 8:**
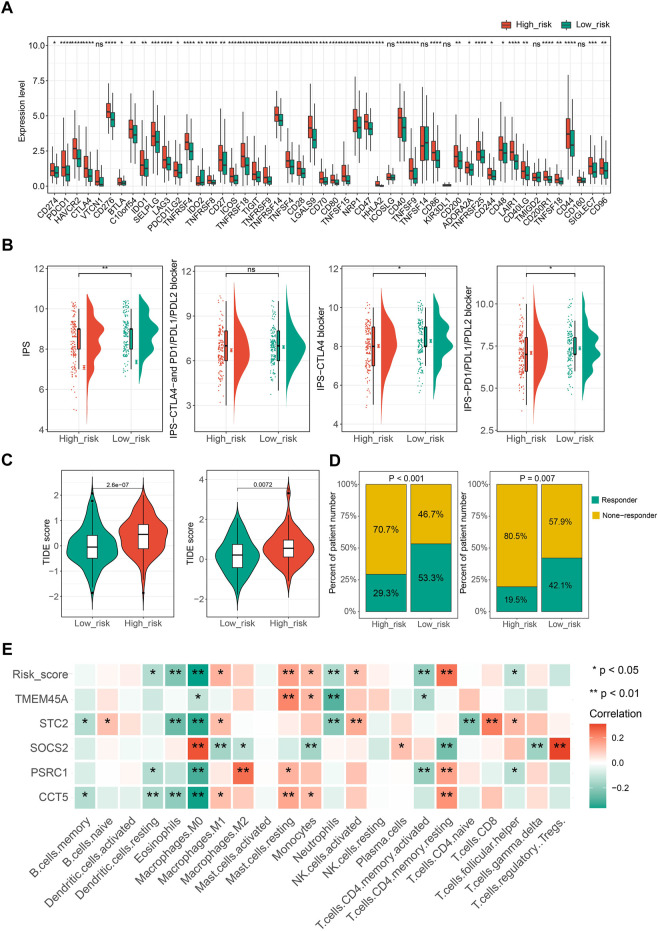
The potential role of CSGscore in predicting immunotherapeutic efficiency and correlation analysis of CSGscore genes and TME cell infiltration. **(A)** The discrepancy of the gene expression of ICGs in two risk groups of the whole TCGA cohort. **(B)** Differences of IPS scores between the two risk groups of the whole TCGA cohort. **(C)** Comparison of TIDE scores between the high- and low-risk groups in the whole TCGA cohort and the ICGC-LIRI-JP dataset. **(D)** The proportions of predicted responders or non-responders in the high- and low-risk groups of the whole TCGA cohort and the ICGC-LIRI-JP dataset. **(E)** The correlation matrix of the five CSGscore genes, CSGscore, and the abundance of 22 immune cell types. *p < 0.05, **p < 0.01, ***p < 0.001, ****p < 0.0001, and ns indicates no significance.

Ongoing efforts are being made to decipher the interplay between cellular senescence and immune response in the tumor microenvironment to determine whether senescence changes the role of the immune system from anti-tumor to pro-tumor response. In this context, we eagerly mined the potential correlation between CSGscore and the tumor immune microenvironment of HCC. We found most of the immune checkpoint genes (ICGs) were highly expressed in the CSGscore-defined high-risk group compared to the low-risk group (Figure 8A). Moreover, Immunophenoscore (IPS), an index to measure the overall immunogenicity of tumors with machine learning, was designed to predict patients’ response to immunocheckpoint inhibitors (ICIs) therapy, and a higher IPS was representative of a more immunogenic tumor. As Figure 8B shows, in the whole TCGA-LIHC cohort, the low-risk patients had higher scores of IPS, IPS-CTLA4, and PD1/PD-L1/PD-L2 blocker than high-risk patients, which demonstrated that low-risk patients had higher sensitivity to ICIs therapy. Also, we performed TIDE scoring on patients in the TCGA-LIHC cohort and the ICGC-LIRI-JP dataset, both of which showed the low-risk patients had significantly lower TIDE scores and a higher proportion of predicted responders (Figures 8C,D). By the CIBERSORT algorithm, we calculated the correlation matrix of the expression levels of the five CSGscore genes and 22 immune cells, respectively. Interestingly, we found that the CSGscore genes were all significantly associated with the M0 macrophage infiltration, positively or negatively in HCC, corresponding to their hazard ratio of them inside the model (Figure 8E). The relative abundance of the 22 immune cell types for the whole TCGA cohort was also shown in Supplementary Figure S5.

In addition, we evaluated the potential application of CSGscore for predicting the HCC patients’ responses to chemo-/targeted therapy. The IC_50_ values of 138 chemo-/targeted drugs were calculated by “pRRophetic” algorithm for HCC patients and 10 common chemo/targeted drugs were selected to show the different IC_50_ values of them in low- and high-CSGscore groups (Supplementary Figure S6).

### 3.7 Comprehensive analysis of the CSGscore genes

**FIGURE 9:**
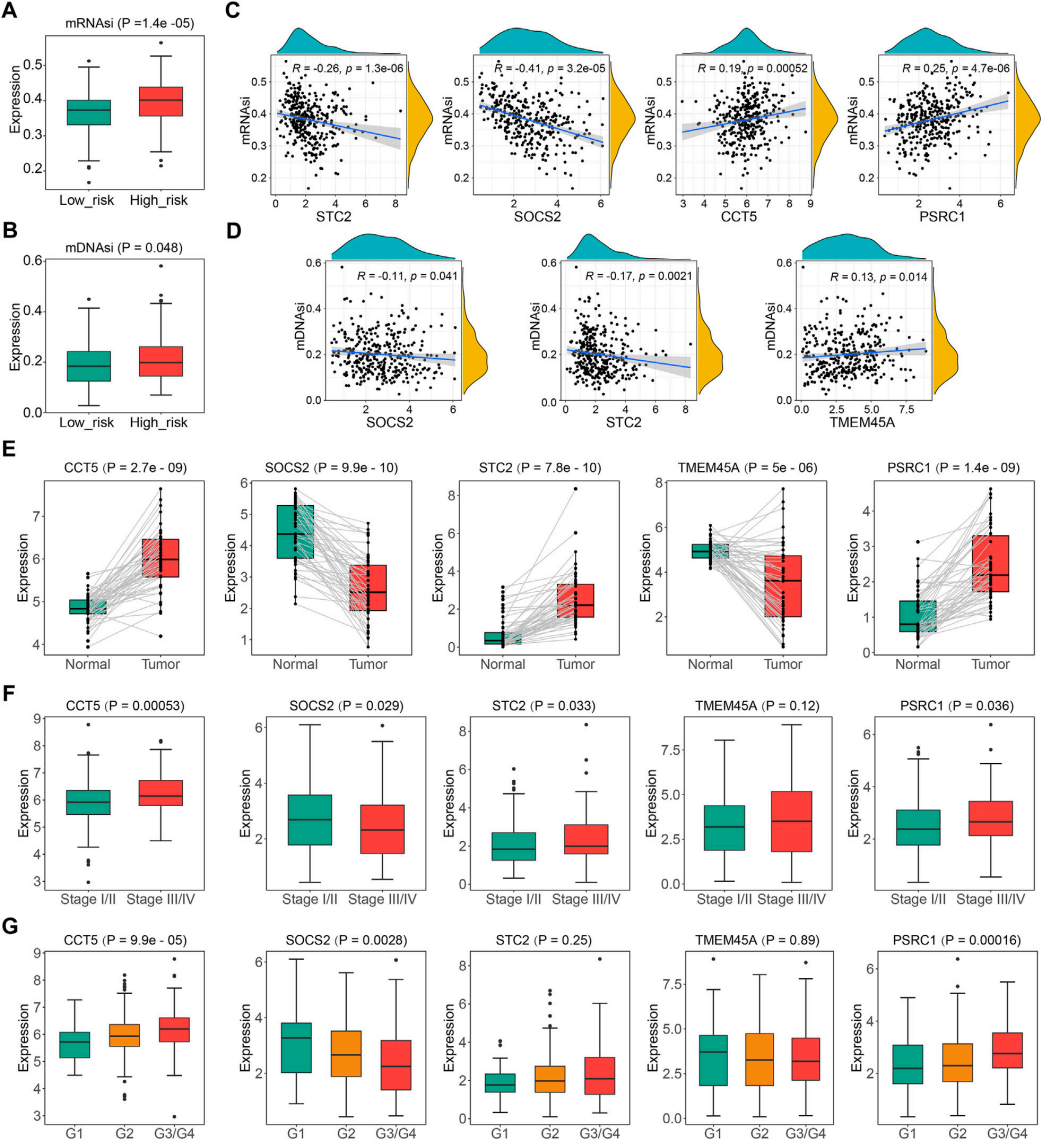
Comprehensive analysis of the five CSGscore genes using the TCGA-LIHC dataset. **(A,B)** Comparison of mRNAsi **(A)** and mDNAsi **(B)** between the high- and low- CSGscore risk groups. **(C,D)** Spearman correlations of the expression of CSGscore genes and mRNAsi **(C)** or mDNAsi **(D)**. Only significant results are shown. **(E)** Validation of the differential expression of the five CSGscore genes between the tumor and normal tissues with paired boxplots. **(F,G)** Comparison of the expression of the five CSGscore genes between subsets of stage and tumor grade.

Significant correlations of cellular senescence levels with tumor stemness have been reported previously (Wang et al., 2022), thus, we suppose the statistical linkage between CSGscore and stemness indices. As shown in Figure 9A and Figure 9B, mRNAsi and mDNAsi both showed significantly different between the low- and high-risk groups, and we noticed that patients in the high-risk group had higher tumor stemness indices, suggesting a worse prognosis in patients with a higher stemness level. Specifically, four CSGscore genes (STC2, SOCS2, CCT5, and PSRC1) were found significantly correlated with mRNAsi while three CSGscore genes (SOCS2, STC2, and TMEM45A) had significant correlations with mDNAsi (Figures 9C,D). Further, we validated the differential expression of the five signature genes with paired boxplots using the TCGA-LIHC dataset (Figure 9E). To seek the clinical relevance of the CSGscore, we analyzed their expression levels according to tumor grade, pathologic stage, tumor status, and CSG subtype. Consequently, most of them showed statistical differences in different subgroups of tumor grade and pathologic stage (Figures 9F,G), implying that they may good indicators for tumor progression. While only TMEM45A was associated with tumor burden status, all of the five signature genes were strongly related to CSG subtypes (Supplementary Figure S7), suggesting their close relationships with the CSG patterns of HCC.

## 4 Discussions

In recent years, numerous studies have identified the carcinogenic properties of cellular senescence, contrary to previous perceptions of it (Ruhland et al., 2016; Calcinotto and Alimonti, 2017). What role cellular senescence plays in the tumorigenesis and development of HCC is far from fully understood. Here, we first identified 238 robust prognostic DECSGs by intersecting DEGs obtained from the TCGA-LIHC cohort with 1,259 CSGs published by a previous study, as well as the “multi-split” selection. Based on these prognostic DECSGs, 336 HCC patients were categorized into two subtypes with distinct survival outcomes and functional characteristics. Subsequently, a CSG subtype predictor was successfully established with two hub genes (TOP2A and KIF11). Then, we proposed a novel CSGs-related prognostic model with five prognostic CSGs: CSGscore, to predict the OS and therapeutic response of HCC.

In the present study, we identified two CSGs-related subtypes of HCC *via* consensus clustering, and subtype 2 exhibited a significantly larger percentage of HCC patients with a high grade or pathologic stage together with a worse survival outcome. GO and KEGG enrichment analysis demonstrated that the most remarkable differences in the functional characteristics of the two subtypes were cell cycle and metabolic pathways, an observation that was strengthened by GSVA and GSEA analysis, both of which revealed that subtype 2 mainly enriched in G2M checkpoint and E2F targets pathways while subtype 1 mainly enriched in multiple metabolism pathways. Thus, the categorization of CSG subtypes corresponds to two dimensions molecularly: cell cycle and metabolism. As well-recognized hallmarks of cancer, cell cycle and metabolism are inextricably linked (Liu et al., 2021b; Wiley and Campisi, 2021). In cancer biology, G2M checkpoint is a hallmark halting the cell cycle in order to repair DNA damage, and its dysregulation is an important contributor to carcinogenesis (Dasika et al., 1999; Bartek and Lukas, 2003). E2F transcription factors and their target genes form a pathway that regulates cell proliferation, and their misexpression leads to the promotion of tumorigenesis (Kent and Leone, 2019). Next, we were prompted to construct a precise and concise predictor for CSGs-related subtypes. With the combination of LASSO regression and random forest, we found that the expression of two key genes (TOP2A and KIF11) could be used to predict the CSG subtype with high accuracies both in the training set and the validation set. Based on the established predictive model, the CSG subtypes for the ICGC-LIRI-JP dataset were further identified, and differential survival outcomes were observed in the two subtypes. TOP2A is an isoform of the Topoisomerase II (TOP2), typically expressing at high levels in rapidly proliferating and growing cells, especially tumor cells, in which overexpression of TOP2A is associated with poor clinical outcomes (Zhao et al., 2008; Liu et al., 2019a). Similar to TOP2A, KIF11, acting as an oncogene that promotes the proliferation of tumor cells, is negatively correlated with decreased overall survival (Jungwirth et al., 2021; Wei et al., 2021a). To this point, we found a novel transcriptional subtype classification of HCC using critical CSGs as the mediators.

To deeply investigate the clinical value of CSGs in HCC, we performed LASSO Cox regression analysis to identify five vital genes (PSRC1, SOCS2, TMEM45A, CCT5, and STC2) from 238 robust prognostic DECSGs, which were subsequently employed to construct the CSGscore risk signature as a scoring system for HCC prognostic prediction. The following validation of the internal and external validation datasets revealed that patients in the high-risk group defined by CSGscore were proved to have a worse prognosis. Among these CSGscore genes, PSRC1, TMEM45A, CCT5, and STC2 were high-risk factors, while SOCS2 was a protective factor. A few studies reported that PSRC1 was a key gene in cancer. For instance, Wei et al. identified PSRC1 as a poor prognostic gene in HCC (Wei et al., 2021b), and Mange et al. (2012) found PSRC1 participating in the progression of breast cancer. In HCC, the expression of PSRC1 was found to be positively associated with cell proliferation and tumor development (Meroni et al., 2021). TMEM45A, a transmembrane protein, which has been proposed as a biomarker of clear cell renal cell carcinoma (ccRCC), was also reported to be associated with chemoresistance in breast cancer and HCC cells (Flamant et al., 2012; Wrzesinski et al., 2015). Recently, a study reported that CCT5 induced epithelial-mesenchymal transition (EMT) to promote gastric cancer lymph node metastasis, and another study reported it promoted lung adenocarcinoma cell migration and invasion (Meng et al., 2021; Li et al., 2022). Similarly, CCT5 overexpression could promote the proliferation, migration, and G1–S transition in HCC cell lines (Liu et al., 2021a). Belonging to a conserved, secreted glycoprotein hormone family, STC2 plays a critical role in regulating the homeostasis of calcium, glucose homeostasis, and phosphorus metastasis (Takei et al., 2012; Qie and Sang, 2022). The dysregulation of STC2 was reported as a major risk factor of EGFR tyrosine kinase inhibitor (TKI) resistance in non-small cell lung cancer (NSCLC) and a predictive marker for lymph node metastasis in esophageal squamous cell carcinoma (Kita et al., 2011; Liu et al., 2019b). Consistent with our study, a remarkable work completed by Chen et al. (2022) found that SOCS2 promoted ferroptosis and radio sensitization in HCC thus improving the prognosis of HCC patients. Importantly, univariate and multivariate analyses confirmed that CSGscore was an independent factor for the prediction of HCC patients’ clinical outcomes. The integrated nomogram, composed of CSGscore, stage, tumor burden, and CSG subtype, was subsequently developed and showed great potential as an ideal quantitative tool to predict 1-, 3-, and 5-year OS in HCC patients.

In advanced HCC, immunotherapies have rapidly evolved in recent years, and most of them focused on monoclonal antibodies against CTLA-4 and PD-1 that block immune checkpoints pathways to reactivate the tumor-killing activity of immune cells in the TME (Cho et al., 2017; Seidel et al., 2018). Although various immunotherapeutic clinical trials have been conducted for HCC, there are still insufficient convincing biomarkers as indicators of therapeutic effects and prognosis. So we explored whether the CSGscore system could classify HCC patients with different intrinsic TME immunity. Encouragingly, the expression levels of almost all ICGs were lower in low-risk HCC patients, indicating that they had a more favorable TIME. IPS and TIDE analyses also validated that the low-risk group had significantly higher sensitivity to anti-CTLA-4 and anti-PD1/PD-L1 immunotherapies. Besides, all five CSGscore genes were found strongly associated with M0 macrophage infiltration in TIME of HCC and the correlation was consistent with the risk coefficient, which might help explain the immune regulatory mechanism for the risk categorization of CSGscore. We virtually computed the IC50 values of 138 chemo/targeted drugs in the two-risk groups and revealed that patients of the low-risk group might respond better to more chemo/targeted drugs than the high-risk group. These results raise the possibility that CSGscore might be a reliable signature in predicting therapeutic response to aid personalized medicine for HCC.

It is well known that cancer stem cells (CSCs) contribute to a high rate of cancer recurrence, as well as resistance to radiotherapy or chemotherapy (Lee et al., 2013). To dig deeper into the performance of the model, we performed correlation analyses between CSGscore and two stemness indices, mRNAsi and mDNAsi, respectively. It turned out that high-risk patients had higher stemness, implying that CSGscore was associated with the stemness in HCC and the signature genes were well worth continued research in the future.

Taken together, we comprehensively evaluated the cellular senescence patterns in HCC and proposed a novel CSG subtype classification system with clinical significance. A CSG subtype predictor was successfully established with high accuracy. In addition, we constructed a novel CSGs-based prognostic signature - CSGscore, which was proved to be a robust and precise indicator for the prediction of OS and therapeutic responses. The correlations of CSGscore genes and immune cell infiltration might advance the dynamic interplay between TIME and cellular senescence in different subgroups of HCC. Finally, the integrative analysis of the CSGscore genes also suggests they are of great importance in the process of tumor progression and represents a promising direction for novel biomarker development of HCC.

## Data Availability

Publicly available datasets were analyzed in this study. This data can be found here: https://portal.gdc.cancer.gov/ and https://icgc.org/.

## Abbreviations

AUCs, areas under the ROC curve; CSGs, cellular senescence-related genes; DCA, decision curve analysis; DECSGs, differentially expressed cellular senescence-related genes; DEGs, differentially expressed genes; DFS, disease-free survival; DSS, disease-specific survival; GSEA, gene set enrichment analysis; GSVA, gene set variation analysis; GO, Gene Ontology; HCC, Hepatocellular carcinoma; IC_50_, semi-inhibitory concentration; ICGC, International Cancer Genome Consortium; ICGs, immune checkpoint genes; ICIs, immunocheckpoint inhibitors; IPS, Immunophenoscore; KEGG, the Kyoto Encyclopedia of Genes and Genomes; LASSO, Least absolute shrinkage and selection operator; OCLR, one-class logistic regression; OS, overall survival; PFS, progression-free survival; ROC, receiver operating characteristic; TIDE, Tumor Immune Dysfunction and Exclusion; TCGA, The Cancer Genome Atlas; TME, tumor microenvironment; TIME, tumor immune microenvironment; tROC, time-dependent receiver operating characteristic; TCIA, The Cancer Immunome Atlas.
