# Supplementary material for: Characterization of cellular senescence patterns predicts the prognosis and therapeutic response of hepatocellular carcinoma
Source: Front Mol Biosci. 2022 Dec 16;9:1100285. doi: 10.3389/fmolb.2022.1100285 (PMC9800843; doi:10.3389/fmolb.2022.1100285)
Supplement: Supplementary file 1 [file DataSheet1.docx]

Supplementary Material

# Supplementary Table 1 Demographic and clinicopathologic information of HCC patients.

| **Characteristics** | **TCGA-LIHC**  **(*n* = 336)** | **ICGC-LIRI-JP**  **(*n* = 238)** |
| --- | --- | --- |
| **Total** | 336 | 238 |
| **Gender** |  |  |
| Female | 108 | 61 |
| Male | 228 | 177 |
| **Age** |  |  |
| <60 | 153 | 49 |
| ≥60 | 183 | 189 |
| **Race** |  |  |
| White | 163 | - |
| Others | 163 | - |
| **BMI** |  |  |
| <25 | 159 | - |
| ≥25 | 150 | - |
| **TNM stage** |  |  |
| I | 157 | 36 |
| II | 77 | 109 |
| III | 79 | 72 |
| VI | 4 | 21 |
| **Grade** |  |  |
| G1 | 52 | - |
| G2 | 157 | - |
| G3 | 110 | - |
| G4 | 12 | - |
| **Progression** |  |  |
| Yes | 161 | 197 |
| No | 175 | 41 |
| **Tumor burden** |  |  |
| Tumor free | 207 | - |
| With tumor | 108 | - |

# Supplementary Table 2 **238 robust prognostic DECSGs**.

| ACTL6A | CKAP2 | ILF2 | NUP37 | STC2 | CENPA | NCAPD2 | CDK1 |
| --- | --- | --- | --- | --- | --- | --- | --- |
| ALYREF | CKAP2L | IMPDH2 | NUSAP1 | STIL | CENPE | NCAPG | FANCI |
| ANLN | CKS1B | IQCC | OIP5 | STMN1 | CENPF | NCAPG2 | MYBL2 |
| ANO10 | CKS2 | KIF11 | ORC1 | TACC3 | CENPH | NCAPH | RFC4 |
| ARHGAP11A | CSE1L | KIF14 | ORC6 | TCERG1 | CENPI | NDC80 | SGOL1 |
| ASF1B | CTSA | KIF15 | PAFAH1B3 | TCF19 | CENPJ | NDRG1 | SOCS2 |
| ASPM | CYSTM1 | KIF18A | PAQR4 | TCF3 | CENPK | NEIL3 | CDKN2A |
| AURKA | DDX11 | KIF18B | PARPBP | TCOF1 | CENPM | NEK2 |  |
| AURKB | DDX39A | KIF20A | PBK | TIMELESS | CENPQ | NPM1 |  |
| BCL2L12 | DEPDC1 | KIF23 | PCK2 | TK1 | CENPW | NRM |  |
| BIRC5 | DEPDC1B | KIF2C | PCNA | TMEM45A | CEP55 | CDKN3 |  |
| BUB1 | DKC1 | KIF4A | PHF19 | TOP2A | CHAF1B | FIGNL1 |  |
| BUB1B | DLGAP5 | KIFC1 | PKMYT1 | TOPBP1 | CHEK1 | RRM1 |  |
| C15orf48 | DNA2 | KNSTRN | PLK1 | TPRKB | GINS1 | SAE1 |  |
| C19orf48 | DNMT1 | KNTC1 | PLK4 | TPX2 | GINS2 | NUDT1 |  |
| CBX3 | DSCC1 | KPNA2 | POC1A | TRIM28 | GLMP | SPC25 |  |
| CCNA2 | DSN1 | LMNB1 | POLA2 | TRIP13 | GTSE1 | CDT1 |  |
| CCNB1 | DTL | LMNB2 | POLD1 | TROAP | H2AFX | FOXM1 |  |
| CCNB2 | DTYMK | MAD2L1 | POLE2 | TTK | H2AFZ | RRM2 |  |
| CCNF | E2F2 | MASP1 | PRC1 | TUBA1B | HAUS1 | SAPCD2 |  |
| CCT5 | E2F8 | MCM10 | PRIM1 | TUBG2 | HELLS | NUF2 |  |
| CDC20 | ECT2 | MCM2 | PRIM2 | TYMS | HJURP | NOP56 |  |
| CDC25B | ESPL1 | MCM3 | PRR11 | UBE2C | HMGA1 | CDKN2B |  |
| CDC25C | ETV4 | MCM4 | PSMC3IP | UBE2S | HMGB2 | FEN1 |  |
| CDC45 | EXO1 | MCM5 | PSMG3 | UBE2T | HMMR | MZT1 |  |
| CDC6 | EZH2 | MCM6 | PSRC1 | UHRF1 | IGFBP3 | RNASEH2A |  |
| CDC7 | FABP5 | MCM7 | PTTG1 | VRK1 | SHCBP1 | NUDCD1 |  |
| CDCA2 | FAM111B | MCM8 | RACGAP1 | WDR76 | SKA1 | SPAG5 |  |
| CDCA3 | FAM219A | MELK | RAD21 | WDYHV1 | SKA3 | FBL |  |
| CDCA4 | FAM83D | MKI67 | RAD51 | ZWINT | SMC4 | MYO19 |  |
| CDCA5 | FANCD2 | MND1 | RAD51AP1 | KIAA0101 | SNRPA | RMI2 |  |
| CDCA7 | FANCE | MSH2 | RAD54L | SGOL2 | SNRPB | NT5DC2 |  |
| CDCA8 | FANCG | MTFR2 | RBBP7 | KIAA1524 | SNRPD1 | SORT1 |  |

# Supplementary Table 3 **208 DEGs between two CSG subtypes of HCC**.

| **Gene symbol** | **logFC** | **AveExpr** | ***t*** | **P.Value** | **adj.P.Val** | **B** |
| --- | --- | --- | --- | --- | --- | --- |
| GINS1 | 2.169627541 | 2.741332253 | 28.47220962 | 1.87E-91 | 3.65E-87 | 198.022222 |
| KIF18B | 2.188121975 | 2.153574958 | 27.78070128 | 6.13E-89 | 5.98E-85 | 192.2614778 |
| TOP2A | 2.751249501 | 4.027768547 | 26.70824805 | 5.48E-85 | 3.56E-81 | 183.2123171 |
| TPX2 | 2.392501976 | 4.42655242 | 26.37352659 | 9.63E-84 | 4.69E-80 | 180.360014 |
| SGOL1 | 1.655564244 | 1.689919642 | 26.27570907 | 2.23E-83 | 8.70E-80 | 179.5240028 |
| KIF11 | 1.872850593 | 2.533360576 | 26.20612027 | 4.06E-83 | 1.32E-79 | 178.928578 |
| BUB1B | 2.076196516 | 2.222380155 | 25.92784742 | 4.47E-82 | 1.24E-78 | 176.5420182 |
| MELK | 2.166560886 | 2.765408791 | 25.81221131 | 1.21E-81 | 2.96E-78 | 175.5476851 |
| CKAP2L | 1.806163514 | 1.86231005 | 25.78299186 | 1.56E-81 | 3.39E-78 | 175.2961928 |
| KIF23 | 1.929268785 | 1.908381528 | 25.76745993 | 1.79E-81 | 3.49E-78 | 175.1624698 |
| KIF4A | 2.247628257 | 2.827315993 | 25.49980927 | 1.82E-80 | 3.22E-77 | 172.8538585 |
| BUB1 | 1.926991984 | 2.210807615 | 25.4248333 | 3.49E-80 | 5.67E-77 | 172.2057215 |
| NCAPG | 2.095296822 | 2.608532449 | 25.2734994 | 1.30E-79 | 1.95E-76 | 170.8956069 |
| LMNB1 | 2.132854058 | 4.204836991 | 25.01135645 | 1.28E-78 | 1.78E-75 | 168.6202696 |
| PLK1 | 2.231916539 | 2.797412668 | 24.95242249 | 2.14E-78 | 2.79E-75 | 168.1077111 |
| KIFC1 | 2.374831823 | 3.592486534 | 24.89169123 | 3.64E-78 | 4.44E-75 | 167.5791305 |
| FANCI | 1.738098072 | 2.519200073 | 24.82960097 | 6.28E-78 | 7.20E-75 | 167.0383133 |
| CDCA8 | 2.164521567 | 3.30052147 | 24.75639698 | 1.19E-77 | 1.29E-74 | 166.4001657 |
| CDK1 | 2.225516242 | 3.393366461 | 24.52783323 | 8.85E-77 | 8.63E-74 | 164.4040424 |
| PRR11 | 1.933715794 | 2.312434032 | 24.52157825 | 9.35E-77 | 8.69E-74 | 164.3493386 |
| KIF2C | 2.272194761 | 2.905574671 | 24.47199497 | 1.45E-76 | 1.28E-73 | 163.9155567 |
| MKI67 | 2.181372647 | 2.839467032 | 24.45090394 | 1.74E-76 | 1.48E-73 | 163.7309633 |
| HJURP | 2.099125157 | 2.680360375 | 24.44449107 | 1.84E-76 | 1.50E-73 | 163.6748273 |
| ARHGAP11A | 1.753837287 | 2.223881029 | 24.42544611 | 2.18E-76 | 1.70E-73 | 163.5080894 |
| NUF2 | 2.143648872 | 2.537092512 | 24.40321162 | 2.65E-76 | 1.99E-73 | 163.3133797 |
| MCM10 | 1.68051071 | 1.557208886 | 24.37927981 | 3.27E-76 | 2.36E-73 | 163.1037493 |
| GTSE1 | 1.970059394 | 2.068355867 | 24.30658393 | 6.20E-76 | 4.32E-73 | 162.4666094 |
| KIF18A | 1.554624709 | 1.390028911 | 24.25443984 | 9.82E-76 | 6.60E-73 | 162.0092612 |
| ZWINT | 2.092104273 | 4.319830402 | 24.21099623 | 1.44E-75 | 9.36E-73 | 161.6280117 |
| ANLN | 2.208876232 | 2.557429538 | 24.1847658 | 1.81E-75 | 1.14E-72 | 161.3977277 |
| FANCD2 | 1.557158949 | 1.874001827 | 24.16082435 | 2.24E-75 | 1.37E-72 | 161.1874783 |
| SPC25 | 1.81023774 | 2.526568087 | 23.98115689 | 1.10E-74 | 6.29E-72 | 159.6078296 |
| TTK | 1.893465285 | 1.980637914 | 23.85286489 | 3.41E-74 | 1.90E-71 | 158.4779079 |
| CENPA | 2.053671599 | 2.317332485 | 23.83414178 | 4.03E-74 | 2.12E-71 | 158.3128697 |
| PRC1 | 2.094654009 | 3.302609305 | 23.79436353 | 5.73E-74 | 2.94E-71 | 157.9621231 |
| CCNF | 1.544387046 | 2.331713814 | 23.7540314 | 8.19E-74 | 4.09E-71 | 157.6063346 |
| TROAP | 2.164087895 | 2.716444927 | 23.69119252 | 1.43E-73 | 6.80E-71 | 157.0516881 |
| SKA3 | 1.829447701 | 2.248300332 | 23.69108109 | 1.43E-73 | 6.80E-71 | 157.0507042 |
| NUSAP1 | 2.130611691 | 4.456435432 | 23.64580229 | 2.14E-73 | 9.92E-71 | 156.6508139 |
| CDC6 | 2.094794534 | 2.916791923 | 23.54760545 | 5.11E-73 | 2.29E-70 | 155.7828881 |
| DLGAP5 | 2.011971308 | 2.389233844 | 23.54615503 | 5.18E-73 | 2.29E-70 | 155.7700615 |
| KIF15 | 1.691836536 | 1.540257755 | 23.42149675 | 1.57E-72 | 6.80E-70 | 154.666912 |
| OIP5 | 1.808469755 | 2.537598162 | 23.380463 | 2.26E-72 | 9.58E-70 | 154.3034677 |
| KIF20A | 2.193670541 | 2.909066353 | 23.27980215 | 5.54E-72 | 2.28E-69 | 153.4112275 |
| NDC80 | 1.891592103 | 2.771639389 | 23.26153155 | 6.52E-72 | 2.56E-69 | 153.2491792 |
| FOXM1 | 2.338641781 | 3.233835237 | 23.17914581 | 1.36E-71 | 5.00E-69 | 152.5180893 |
| RAD54L | 1.783739876 | 1.597902369 | 23.17191651 | 1.45E-71 | 5.23E-69 | 152.4539068 |
| NCAPH | 1.960495507 | 2.530803296 | 23.00087517 | 6.67E-71 | 2.28E-68 | 150.9340007 |
| NEK2 | 2.154044005 | 2.928610119 | 22.96006573 | 9.60E-71 | 3.23E-68 | 150.5709712 |
| DEPDC1 | 1.790414777 | 1.720704919 | 22.95280153 | 1.02E-70 | 3.39E-68 | 150.5063354 |
| CCNB2 | 2.194164519 | 3.316805827 | 22.9458815 | 1.09E-70 | 3.54E-68 | 150.4447575 |
| KIAA1524 | 1.608435808 | 1.615217276 | 22.94371095 | 1.11E-70 | 3.55E-68 | 150.425442 |
| MCM2 | 2.176054986 | 4.206494973 | 22.93395566 | 1.21E-70 | 3.81E-68 | 150.3386255 |
| ORC1 | 1.765192897 | 2.132994915 | 22.89772633 | 1.68E-70 | 5.19E-68 | 150.0161312 |
| HELLS | 1.526317959 | 1.644301938 | 22.88762248 | 1.83E-70 | 5.59E-68 | 149.9261715 |
| ECT2 | 2.034310051 | 2.858353994 | 22.87374291 | 2.08E-70 | 6.23E-68 | 149.8025799 |
| CCNB1 | 2.201707206 | 4.473881685 | 22.86688555 | 2.21E-70 | 6.53E-68 | 149.7415117 |
| CDCA5 | 1.980141858 | 3.228224321 | 22.83393858 | 2.97E-70 | 8.63E-68 | 149.4480453 |
| RFC4 | 1.559729746 | 4.047357057 | 22.81036376 | 3.66E-70 | 1.05E-67 | 149.2380005 |
| E2F8 | 1.73948663 | 1.679543977 | 22.80611307 | 3.80E-70 | 1.08E-67 | 149.2001229 |
| RAD51 | 1.589802545 | 2.025937065 | 22.73342894 | 7.29E-70 | 2.01E-67 | 148.5521969 |
| MYBL2 | 2.93602408 | 3.950260057 | 22.73291421 | 7.33E-70 | 2.01E-67 | 148.5476068 |
| ASF1B | 2.149364879 | 3.4471155 | 22.72916007 | 7.58E-70 | 2.05E-67 | 148.5141288 |
| SKA1 | 2.030818648 | 2.432212905 | 22.70160363 | 9.70E-70 | 2.59E-67 | 148.2683536 |
| EXO1 | 1.729552172 | 2.066491749 | 22.65052272 | 1.53E-69 | 4.04E-67 | 147.8125918 |
| DEPDC1B | 2.091946112 | 2.112139748 | 22.63846433 | 1.71E-69 | 4.44E-67 | 147.70497 |
| CDC45 | 1.894062777 | 2.731414726 | 22.60860311 | 2.23E-69 | 5.73E-67 | 147.4384038 |
| CENPF | 2.105693236 | 2.64294696 | 22.51004547 | 5.40E-69 | 1.37E-66 | 146.5580609 |
| UHRF1 | 1.960248203 | 2.0173727 | 22.44492098 | 9.70E-69 | 2.42E-66 | 145.9759054 |
| RAD51AP1 | 1.822440495 | 2.406360605 | 22.4059708 | 1.38E-68 | 3.40E-66 | 145.6275571 |
| CDCA2 | 1.546841187 | 1.438243172 | 22.31591376 | 3.09E-68 | 7.44E-66 | 144.8216616 |
| EZH2 | 1.627657549 | 3.135010771 | 22.19356582 | 9.29E-68 | 2.18E-65 | 143.7257519 |
| KIAA0101 | 1.838745965 | 2.913349217 | 22.07169017 | 2.79E-67 | 6.32E-65 | 142.6328899 |
| RRM2 | 2.112006581 | 3.998058927 | 21.9350647 | 9.55E-67 | 2.09E-64 | 141.4063934 |
| CDCA3 | 1.679389998 | 2.210810642 | 21.74748651 | 5.19E-66 | 1.10E-63 | 139.7201905 |
| DTL | 1.983751036 | 2.79393793 | 21.637262 | 1.41E-65 | 2.92E-63 | 138.7281384 |
| CDC20 | 2.570400071 | 4.322459247 | 21.58149763 | 2.33E-65 | 4.78E-63 | 138.2259109 |
| CEP55 | 1.888275861 | 2.026564673 | 21.48624836 | 5.52E-65 | 1.12E-62 | 137.3675657 |
| RACGAP1 | 1.742020233 | 3.406806539 | 21.45673232 | 7.20E-65 | 1.45E-62 | 137.1014518 |
| KNTC1 | 1.580237873 | 2.063125609 | 21.36702185 | 1.62E-64 | 3.23E-62 | 136.2922644 |
| TRIP13 | 1.889979162 | 2.301395691 | 21.34409718 | 2.00E-64 | 3.94E-62 | 136.0853968 |
| BIRC5 | 2.32174372 | 3.933255532 | 21.21692112 | 6.33E-64 | 1.22E-61 | 134.9371514 |
| PBK | 2.043712381 | 2.9416017 | 21.1530017 | 1.13E-63 | 2.14E-61 | 134.3596378 |
| MAD2L1 | 1.585759683 | 2.341837546 | 20.92094194 | 9.31E-63 | 1.71E-60 | 132.2608105 |
| TYMS | 1.981941784 | 4.472345878 | 20.89969005 | 1.13E-62 | 2.04E-60 | 132.0684366 |
| STMN1 | 1.77937944 | 5.117579696 | 20.89099814 | 1.22E-62 | 2.19E-60 | 131.9897489 |
| MCM6 | 1.699268146 | 4.511179393 | 20.8504362 | 1.77E-62 | 3.13E-60 | 131.6224832 |
| CCNA2 | 2.155850783 | 3.476254663 | 20.7068091 | 6.53E-62 | 1.15E-59 | 130.3212514 |
| CDT1 | 2.046205119 | 3.344082197 | 20.70583402 | 6.59E-62 | 1.15E-59 | 130.3124135 |
| ASPM | 1.890379144 | 2.523719334 | 20.56009606 | 2.48E-61 | 4.21E-59 | 128.9908655 |
| CHAF1B | 1.75090406 | 2.111077487 | 20.5129731 | 3.82E-61 | 6.41E-59 | 128.5633098 |
| CDC25A | 1.562484762 | 1.884337023 | 20.41944962 | 8.95E-61 | 1.49E-58 | 127.7144092 |
| TCF19 | 2.009060638 | 3.822044978 | 20.34571641 | 1.75E-60 | 2.90E-58 | 127.0448277 |
| CDC25C | 1.748938753 | 2.320698742 | 20.30853376 | 2.46E-60 | 4.03E-58 | 126.7070645 |
| WDR76 | 1.694955035 | 2.444997288 | 20.27738064 | 3.27E-60 | 5.31E-58 | 126.4240209 |
| CENPM | 2.096895695 | 3.335166063 | 20.20093381 | 6.57E-60 | 1.05E-57 | 125.7292617 |
| CDC7 | 1.526481817 | 2.163674071 | 20.12561895 | 1.31E-59 | 2.07E-57 | 125.0445235 |
| KPNA2 | 1.552596664 | 5.791456158 | 19.94103047 | 7.06E-59 | 1.10E-56 | 123.3652394 |
| HMMR | 1.841877018 | 2.797114087 | 19.83251842 | 1.90E-58 | 2.90E-56 | 122.3773903 |
| TACC3 | 1.730487321 | 3.675173348 | 19.8154804 | 2.22E-58 | 3.36E-56 | 122.2222406 |
| NCAPD2 | 1.622849011 | 3.829843435 | 19.73147617 | 4.80E-58 | 7.14E-56 | 121.4571284 |
| PKMYT1 | 1.572011986 | 2.130215264 | 19.57292818 | 2.05E-57 | 2.98E-55 | 120.0123694 |
| SHCBP1 | 1.659713738 | 1.735048368 | 19.53269466 | 2.96E-57 | 4.27E-55 | 119.6456064 |
| SPAG5 | 1.690472389 | 3.96600853 | 19.49590958 | 4.14E-57 | 5.94E-55 | 119.3102326 |
| AURKB | 2.024803478 | 3.264458182 | 19.37871118 | 1.21E-56 | 1.70E-54 | 118.2414398 |
| UBE2T | 1.817019574 | 4.553766619 | 19.33814389 | 1.76E-56 | 2.45E-54 | 117.8713905 |
| HMGB2 | 1.60560049 | 5.576650098 | 19.26394759 | 3.47E-56 | 4.73E-54 | 117.1944628 |
| MCM3 | 1.617613014 | 5.851647369 | 19.25386245 | 3.80E-56 | 5.15E-54 | 117.1024399 |
| CDKN3 | 2.036229943 | 3.691375122 | 19.08296397 | 1.82E-55 | 2.40E-53 | 115.5426716 |
| UBE2C | 2.432384977 | 4.691046537 | 19.00511976 | 3.72E-55 | 4.80E-53 | 114.8319753 |
| MCM4 | 1.652968346 | 4.375046054 | 18.85847836 | 1.43E-54 | 1.83E-52 | 113.4928537 |
| DNMT1 | 1.549531549 | 3.680321342 | 18.81049714 | 2.22E-54 | 2.77E-52 | 113.0546098 |
| RMI2 | 1.76875189 | 2.897561609 | 18.78882494 | 2.70E-54 | 3.36E-52 | 112.8566516 |
| C16orf59 | 1.599625263 | 2.47806441 | 18.62989126 | 1.16E-53 | 1.38E-51 | 111.4047209 |
| E2F1 | 2.154432301 | 4.326732242 | 18.19588435 | 6.23E-52 | 7.03E-50 | 107.4387821 |
| PTTG1 | 2.050299738 | 4.342275267 | 17.68689458 | 6.66E-50 | 6.94E-48 | 102.7880255 |
| CENPW | 1.746333357 | 4.348145557 | 17.41830178 | 7.82E-49 | 7.70E-47 | 100.335211 |
| CENPU | 1.642480501 | 3.323164432 | 17.40782604 | 8.61E-49 | 8.44E-47 | 100.2395765 |
| ESPL1 | 1.552948196 | 2.124137553 | 17.16443509 | 8.01E-48 | 7.62E-46 | 98.01845068 |
| FAM111B | 1.687514233 | 2.603216649 | 16.92558153 | 7.14E-47 | 6.51E-45 | 95.84057523 |
| NRM | 1.622631059 | 4.215297836 | 16.56960179 | 1.85E-45 | 1.61E-43 | 92.59912114 |
| SPC24 | 1.690214349 | 3.535318094 | 16.37037159 | 1.14E-44 | 9.74E-43 | 90.78778572 |
| RIBC2 | 1.549338577 | 1.398821126 | 16.24034021 | 3.75E-44 | 3.09E-42 | 89.60684379 |
| TK1 | 1.731816503 | 5.630489467 | 16.2114272 | 4.88E-44 | 3.99E-42 | 89.34440198 |
| AURKA | 1.556603331 | 4.184744997 | 15.69803968 | 5.21E-42 | 3.91E-40 | 84.6944203 |
| SMC4 | 1.66379196 | 2.760651023 | 15.43897923 | 5.46E-41 | 3.83E-39 | 82.35624492 |
| CCNE1 | 1.84286354 | 2.373399807 | 14.40107485 | 6.23E-37 | 3.62E-35 | 73.06119324 |
| TUBA1B | 1.629136367 | 6.16770347 | 13.99453622 | 2.33E-35 | 1.20E-33 | 69.45967424 |
| FAM83D | 1.566349792 | 3.847000786 | 13.64085181 | 5.32E-34 | 2.48E-32 | 66.34828828 |
| PAQR4 | 1.53703037 | 2.863707978 | 13.61310973 | 6.79E-34 | 3.12E-32 | 66.10517249 |
| IQGAP3 | 1.525507767 | 2.945331976 | 13.14934834 | 3.96E-32 | 1.57E-30 | 62.06284576 |
| CDCA7 | 1.609318161 | 1.436248343 | 11.83706725 | 3.04E-27 | 7.45E-26 | 50.88723614 |
| G6PD | 1.69883582 | 4.561568372 | 11.73824189 | 6.97E-27 | 1.65E-25 | 50.06394112 |
| PKM | 1.774846934 | 5.310079735 | 11.61383341 | 1.97E-26 | 4.44E-25 | 49.0316116 |
| PAFAH1B3 | 1.538025963 | 4.854700655 | 11.5137439 | 4.53E-26 | 9.76E-25 | 48.20446919 |
| DSG2 | 1.79770406 | 3.802843241 | 11.32705447 | 2.13E-25 | 4.25E-24 | 46.66998349 |
| NT5DC2 | 1.533049671 | 3.659340976 | 11.03089091 | 2.42E-24 | 4.25E-23 | 44.25883334 |
| C12orf75 | 1.879854568 | 3.152965316 | 10.9088736 | 6.52E-24 | 1.08E-22 | 43.27406677 |
| IGSF3 | 1.55542552 | 2.465798912 | 10.88620494 | 7.84E-24 | 1.29E-22 | 43.09168455 |
| CFHR4 | -2.150679097 | 4.374590832 | -10.87850947 | 8.34E-24 | 1.36E-22 | 43.02981119 |
| CLGN | 1.836854445 | 2.990122591 | 10.18038584 | 2.19E-21 | 2.72E-20 | 37.50745143 |
| C1orf106 | 1.517454048 | 1.659855179 | 9.797359412 | 4.30E-20 | 4.59E-19 | 34.55923942 |
| SLC1A5 | 1.524764383 | 3.946154214 | 9.760340502 | 5.71E-20 | 6.00E-19 | 34.27757679 |
| SLC27A5 | -1.851821517 | 6.495409917 | -9.71227497 | 8.26E-20 | 8.49E-19 | 33.91275042 |
| NRSN2 | 1.583814377 | 3.272970872 | 9.694665199 | 9.45E-20 | 9.66E-19 | 33.77934094 |
| ASPDH | -1.832677716 | 5.447381697 | -9.266558031 | 2.40E-18 | 2.06E-17 | 30.57881193 |
| CA9 | 2.220075174 | 1.847039158 | 9.23809324 | 2.97E-18 | 2.51E-17 | 30.3689995 |
| HPR | -1.985049792 | 8.270088523 | -9.09774225 | 8.39E-18 | 6.76E-17 | 29.34011214 |
| HPD | -2.780569077 | 8.990498151 | -9.00840974 | 1.62E-17 | 1.26E-16 | 28.6901774 |
| ELOVL7 | 1.582508426 | 2.027776183 | 8.969819518 | 2.15E-17 | 1.65E-16 | 28.41062288 |
| SPHK1 | 1.729802192 | 2.713841847 | 8.940084808 | 2.67E-17 | 2.02E-16 | 28.19572014 |
| GAL3ST1 | 1.874536955 | 2.949799239 | 8.927580922 | 2.93E-17 | 2.19E-16 | 28.10548112 |
| IGF2BP2 | 1.59996326 | 2.85716787 | 8.832857361 | 5.83E-17 | 4.21E-16 | 27.42440536 |
| DUSP9 | 1.830363876 | 3.37355097 | 8.746543286 | 1.09E-16 | 7.60E-16 | 26.80773074 |
| HP | -1.998178933 | 11.03369578 | -8.678718459 | 1.77E-16 | 1.21E-15 | 26.32582044 |
| AKR7A3 | -1.843177659 | 6.002411254 | -8.591862033 | 3.30E-16 | 2.18E-15 | 25.71215622 |
| ACSM2A | -1.594180648 | 5.586080226 | -8.582512129 | 3.53E-16 | 2.32E-15 | 25.64633086 |
| ADH1B | -2.079259593 | 8.303040805 | -8.428824071 | 1.05E-15 | 6.58E-15 | 24.5709407 |
| MASP2 | -1.660261852 | 6.432444167 | -8.141530404 | 7.78E-15 | 4.40E-14 | 22.59484031 |
| AQP9 | -2.113597647 | 7.392620179 | -8.022687764 | 1.76E-14 | 9.54E-14 | 21.790772 |
| TTC36 | -2.021375653 | 3.656602816 | -8.014227327 | 1.86E-14 | 1.01E-13 | 21.73383444 |
| C6 | -1.551207407 | 6.613977936 | -7.87690553 | 4.73E-14 | 2.45E-13 | 20.81540382 |
| SLC29A4 | 1.539701714 | 2.584725734 | 7.836353982 | 6.22E-14 | 3.18E-13 | 20.54626935 |
| SLC10A1 | -2.203889507 | 6.198408378 | -7.65685471 | 2.06E-13 | 1.00E-12 | 19.36654272 |
| PON1 | -1.646647085 | 7.598756005 | -7.651677074 | 2.13E-13 | 1.03E-12 | 19.33279691 |
| PEG10 | 2.475026919 | 3.446969717 | 7.608567821 | 2.83E-13 | 1.35E-12 | 19.05245052 |
| TAT | -2.295866673 | 7.195537361 | -7.597604415 | 3.05E-13 | 1.45E-12 | 18.98133161 |
| GLYAT | -2.021348012 | 4.768358095 | -7.54209861 | 4.39E-13 | 2.06E-12 | 18.62238147 |
| CD24 | 2.005843747 | 5.756614292 | 7.47513084 | 6.80E-13 | 3.11E-12 | 18.19179555 |
| UPK3A | 1.929641347 | 2.188667085 | 7.439508972 | 8.57E-13 | 3.88E-12 | 17.96387196 |
| TRNP1 | 1.557476226 | 3.926836367 | 7.438533451 | 8.63E-13 | 3.90E-12 | 17.95764112 |
| MEP1A | 1.528034604 | 1.456537028 | 7.420180431 | 9.72E-13 | 4.36E-12 | 17.84052596 |
| SERPINC1 | -1.740638969 | 11.5002438 | -7.247373475 | 2.95E-12 | 1.25E-11 | 16.7480446 |
| CYP4A11 | -1.50235365 | 7.124258247 | -7.147689136 | 5.56E-12 | 2.31E-11 | 16.1263699 |
| HFE2 | -1.537890301 | 7.440643671 | -7.133227595 | 6.09E-12 | 2.52E-11 | 16.03670597 |
| SLC13A5 | -1.563776128 | 5.606687037 | -7.114590561 | 6.85E-12 | 2.82E-11 | 15.92135039 |
| VIL1 | 1.591986721 | 3.807352381 | 7.087362359 | 8.13E-12 | 3.31E-11 | 15.75321901 |
| CYP3A4 | -2.862585376 | 6.340548356 | -7.082403989 | 8.39E-12 | 3.42E-11 | 15.7226528 |
| ADH4 | -2.312384337 | 7.767498596 | -7.076129003 | 8.72E-12 | 3.55E-11 | 15.68399289 |
| PYCR1 | 1.542749957 | 3.459794215 | 6.981133418 | 1.58E-11 | 6.27E-11 | 15.1018356 |
| APOA5 | -1.526821477 | 7.638278017 | -6.923998757 | 2.25E-11 | 8.80E-11 | 14.75452455 |
| GYS2 | -1.580288554 | 4.315698036 | -6.842073374 | 3.72E-11 | 1.42E-10 | 14.26025303 |
| F9 | -1.747346629 | 6.641205341 | -6.840651065 | 3.76E-11 | 1.44E-10 | 14.25171108 |
| CFHR3 | -1.618696525 | 5.164025781 | -6.805438739 | 4.66E-11 | 1.76E-10 | 14.04066436 |
| AFP | 2.385380077 | 4.274964882 | 6.672003348 | 1.05E-10 | 3.80E-10 | 13.24842361 |
| AOX1 | -1.532800119 | 7.675388692 | -6.60999594 | 1.52E-10 | 5.42E-10 | 12.88435087 |
| ADH1C | -1.967057733 | 8.096323092 | -6.576811865 | 1.85E-10 | 6.55E-10 | 12.69058591 |
| CYP2C9 | -1.630329887 | 7.470952062 | -6.55165856 | 2.15E-10 | 7.55E-10 | 12.54421428 |
| APOF | -1.546219772 | 5.610929797 | -6.416746355 | 4.75E-10 | 1.61E-09 | 11.76656193 |
| BEX2 | 1.534919257 | 2.832535154 | 6.350103171 | 7.01E-10 | 2.34E-09 | 11.38708044 |
| SLC22A1 | -1.943423038 | 6.078102084 | -6.217664655 | 1.50E-09 | 4.85E-09 | 10.64220467 |
| CTAG2 | 1.535104733 | 1.460379848 | 6.170617179 | 1.96E-09 | 6.27E-09 | 10.38058732 |
| HSD11B1 | -1.975406249 | 7.298438573 | -6.071542207 | 3.43E-09 | 1.07E-08 | 9.834842411 |
| PCK1 | -1.636356426 | 6.90161858 | -5.928208612 | 7.62E-09 | 2.29E-08 | 9.057869518 |
| HGFAC | -1.811681361 | 4.480624852 | -5.925488074 | 7.73E-09 | 2.32E-08 | 9.043267167 |
| KRT19 | 1.5783829 | 2.418003972 | 5.884080133 | 9.71E-09 | 2.88E-08 | 8.821683045 |
| CYP8B1 | -1.880649208 | 6.048191365 | -5.868994086 | 1.05E-08 | 3.12E-08 | 8.741267458 |
| SLC25A47 | -1.84294117 | 5.398220379 | -5.868966594 | 1.05E-08 | 3.12E-08 | 8.741121066 |
| UGT1A4 | -1.693773858 | 4.464548419 | -5.852582643 | 1.15E-08 | 3.39E-08 | 8.653977577 |
| SAA1 | -2.025331741 | 8.83104197 | -5.569624592 | 5.25E-08 | 1.44E-07 | 7.18054931 |
| CYP2E1 | -2.160414887 | 7.142717536 | -5.472692831 | 8.70E-08 | 2.34E-07 | 6.689714013 |
| SPP2 | -1.521761887 | 6.329606687 | -5.461851054 | 9.20E-08 | 2.47E-07 | 6.635261367 |
| CYP1A2 | -1.76171831 | 2.954590262 | -5.448267211 | 9.87E-08 | 2.64E-07 | 6.567164189 |
| CYP2A6 | -2.129538597 | 7.122795611 | -5.425408009 | 1.11E-07 | 2.96E-07 | 6.452889166 |
| SAA2-SAA4 | -1.613290885 | 4.357426256 | -5.21057472 | 3.30E-07 | 8.31E-07 | 5.398720854 |
| SAA2 | -1.660046113 | 5.610595983 | -4.816728882 | 2.21E-06 | 5.08E-06 | 3.560931596 |
| SPP1 | 1.695023834 | 7.09763371 | 4.55527234 | 7.34E-06 | 1.59E-05 | 2.410397562 |
| SDS | -1.542091146 | 6.581411389 | -4.475882422 | 1.04E-05 | 2.22E-05 | 2.072246797 |

**Supplementary Figures**


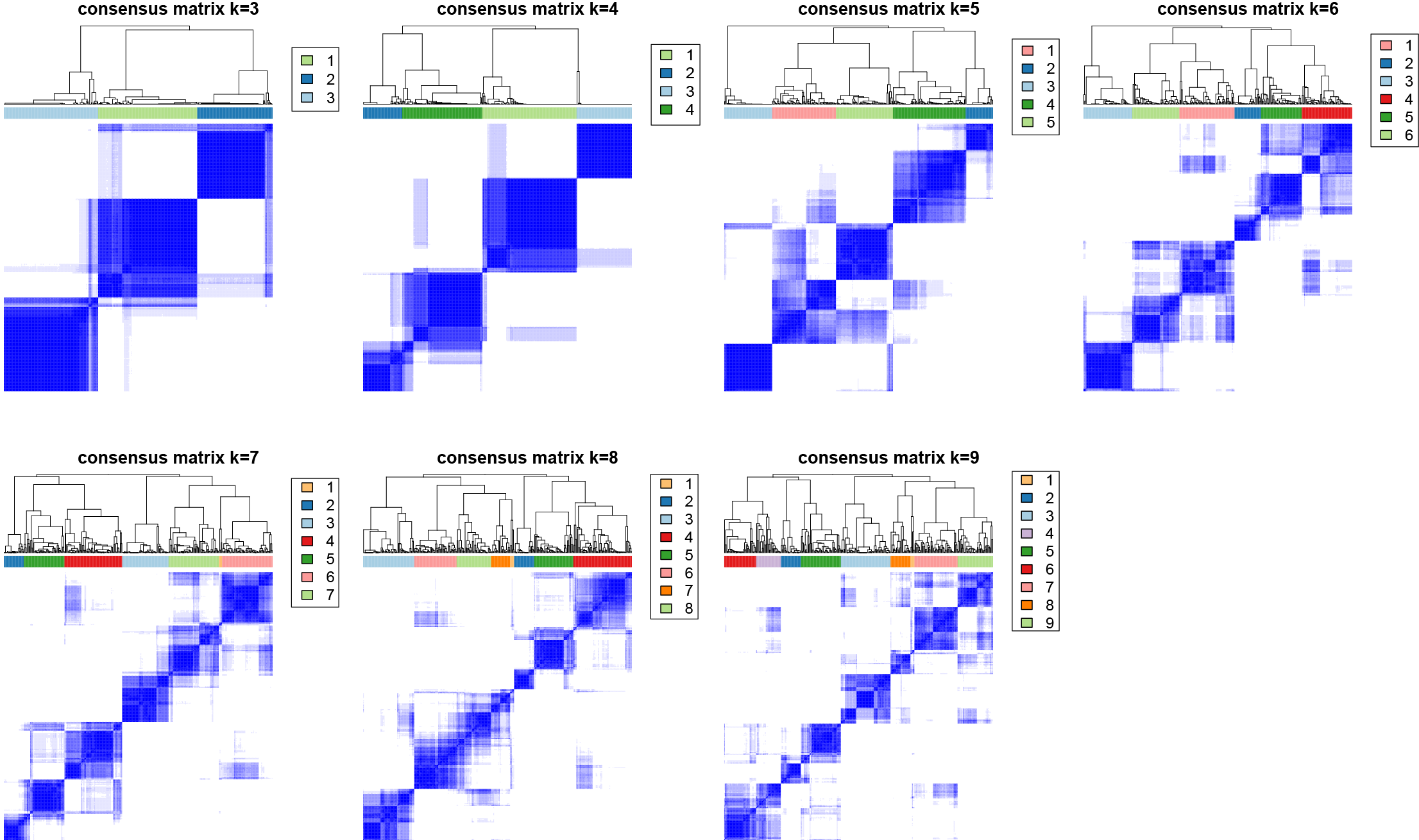


**Supplementary Figure 1 Unsupervised clustering of the 238 robust prognostic DECSGs and consensus matrix heatmaps for k = 3-9.**

.


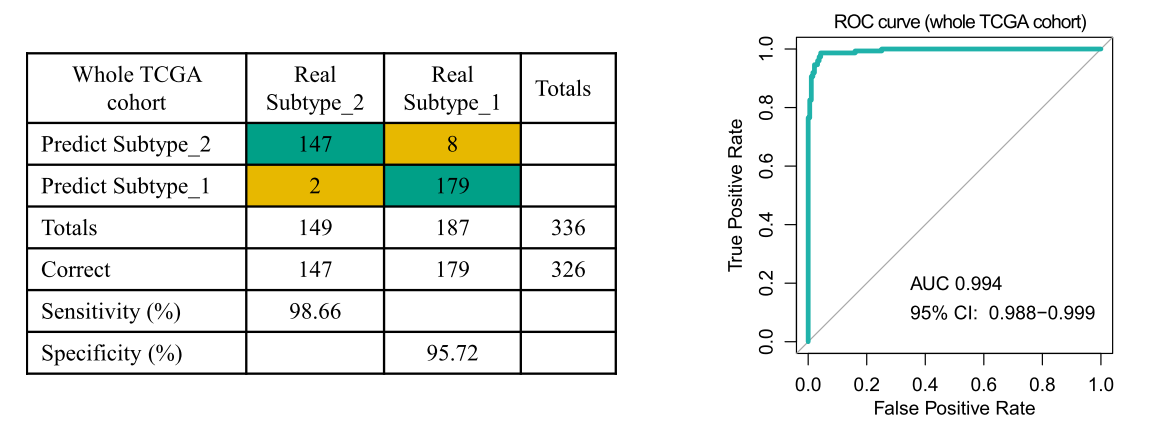


**Supplementary Figure 2 Confusion matrix and ROC curve of the CSG subtype predictor for the binary classification of the whole TCGA-LIHC cohort.**


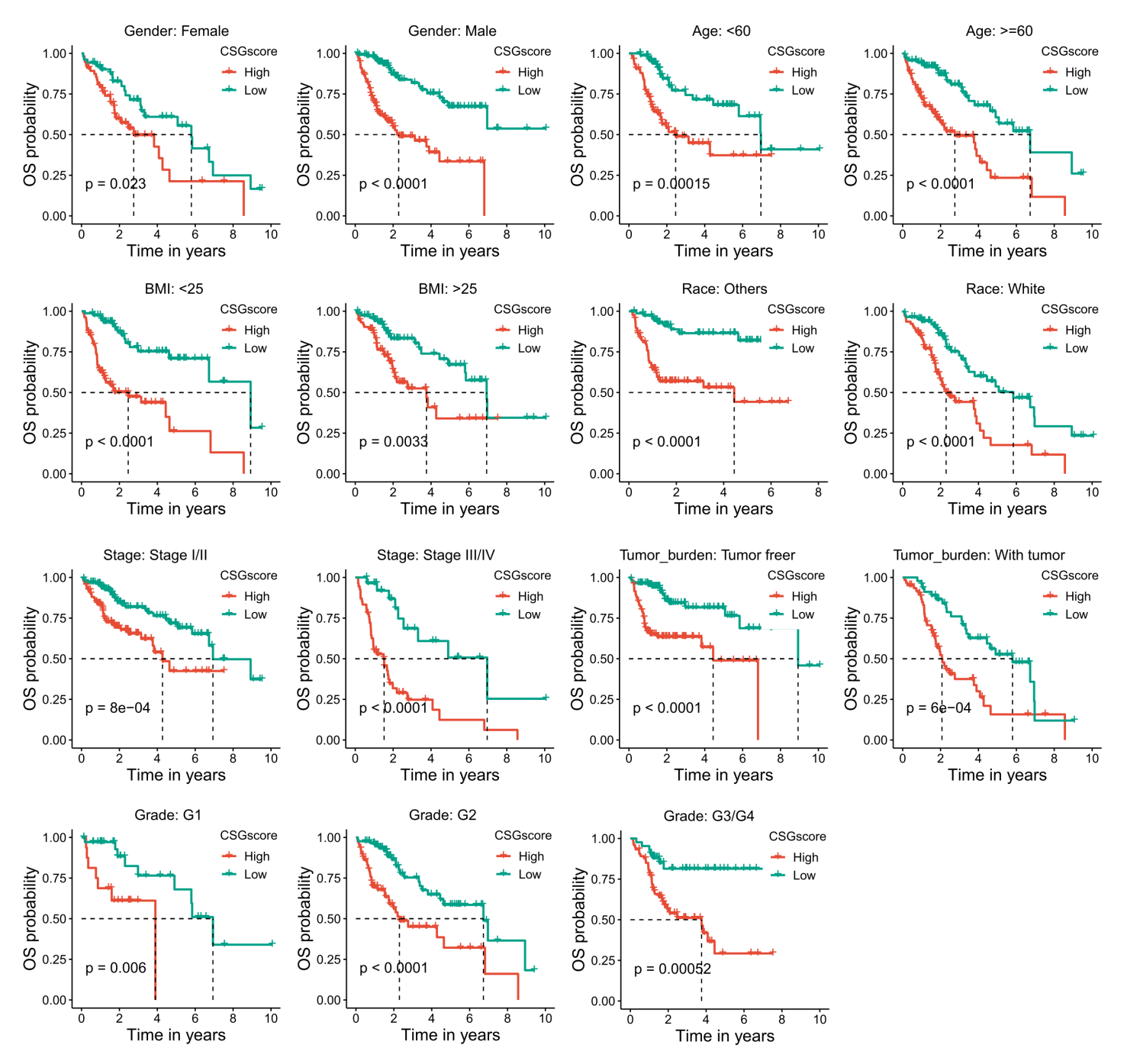


**Supplementary Figure 3 Stratified survival analysis of the CSGscore system in subsets divided by clinicopathologic features.**


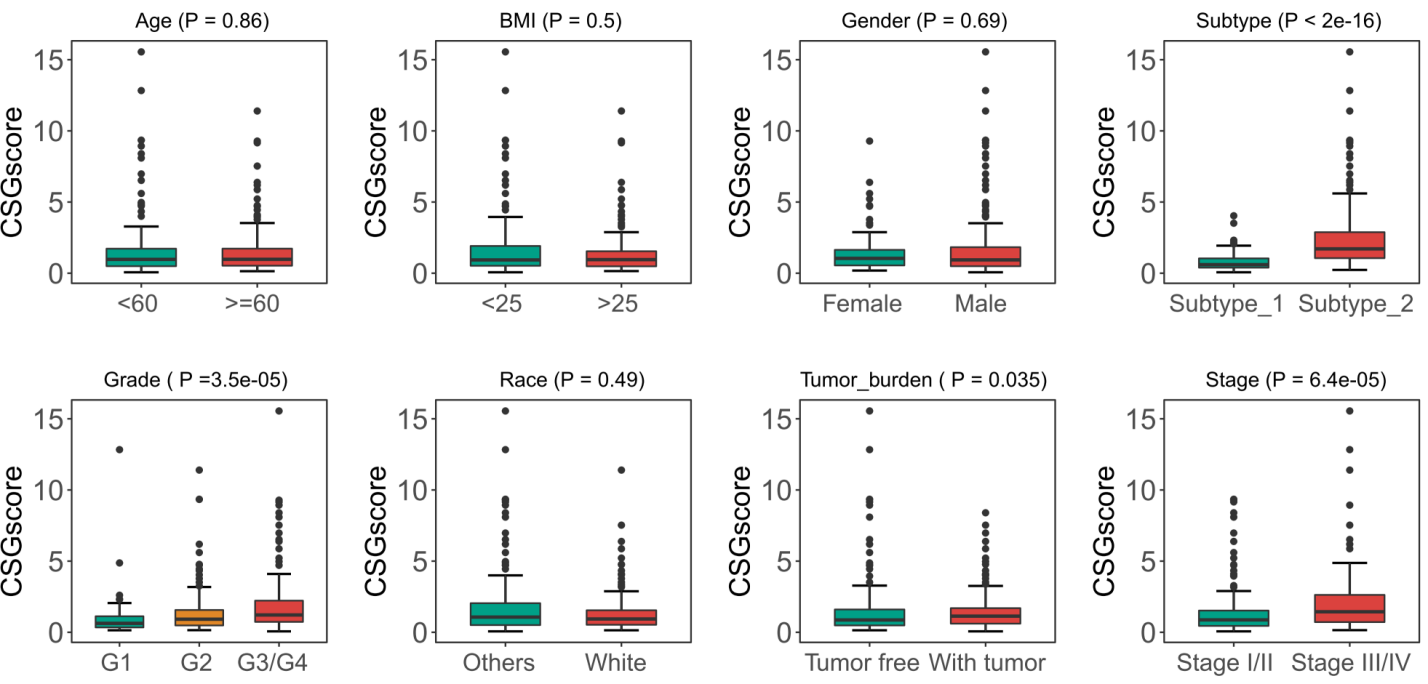


**Supplementary Figure 4 Boxplots showing the correlations of CSGscore and clinicopathologic parameters.**


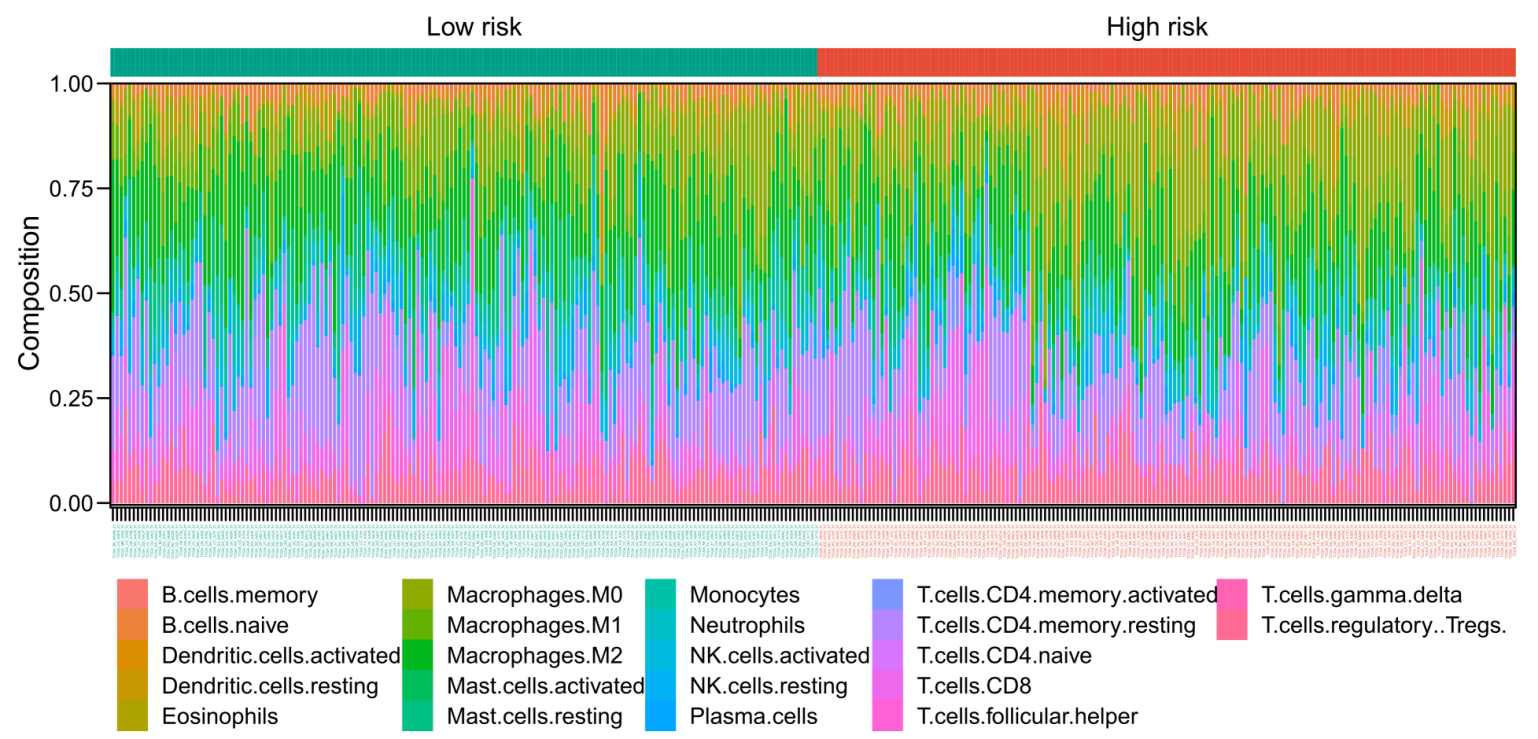


**Supplementary Figure 5 TME cell landscape of the whole TCGA-LIHC cohort computed by the CIBERSORT algorithm.**


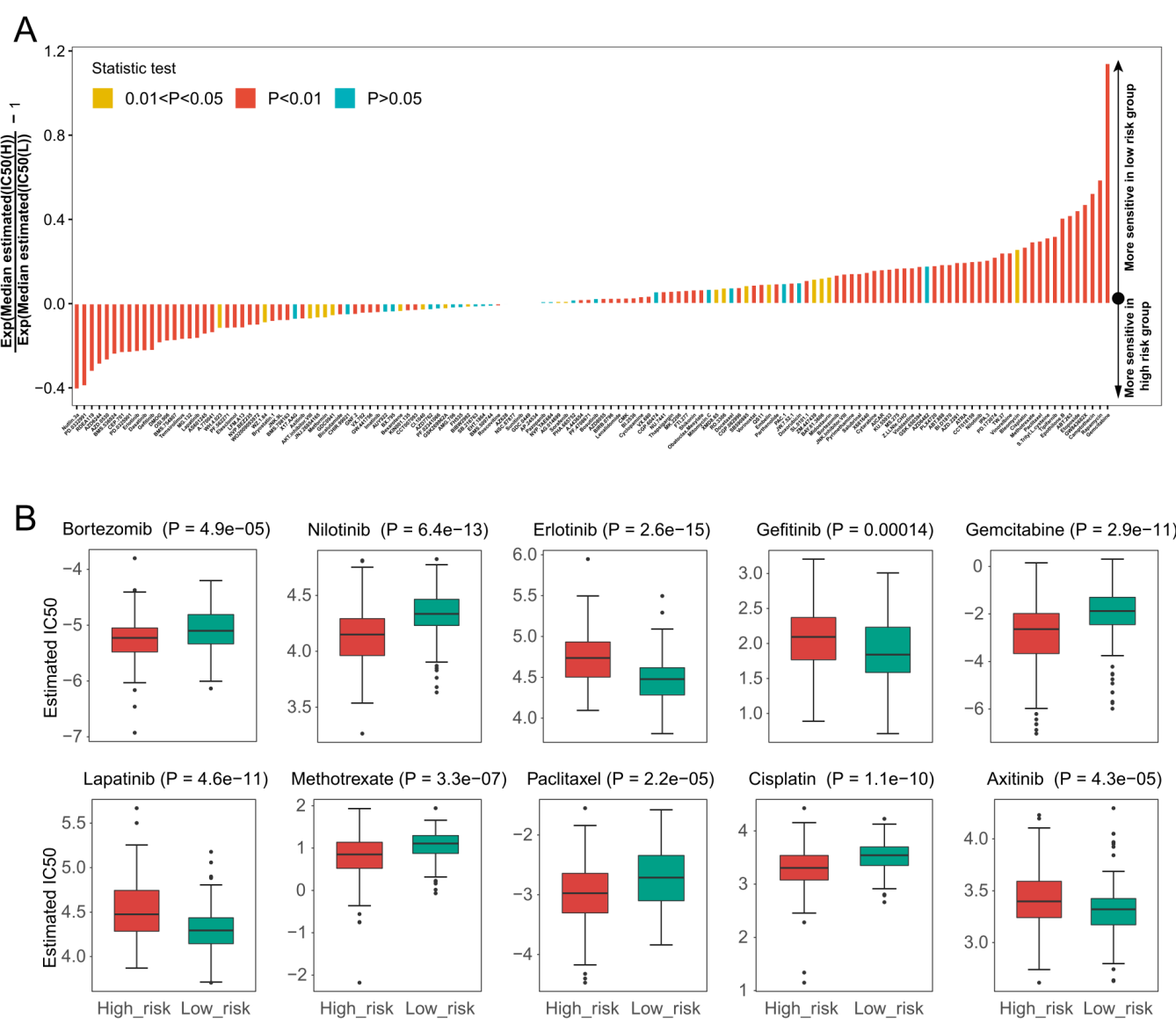


**Supplementary Figure 6 Prediction performance of CSGscore for the chemo/targeted therapy sensitivities.** (A) Estimation of the normalized IC_50_ values of 138 chemo/targeted therapeutic drugs. (B) Boxplots showing the differential estimated IC50 of common chemo/targeted therapeutic drugs in the high- or low- CSGscore risk groups.


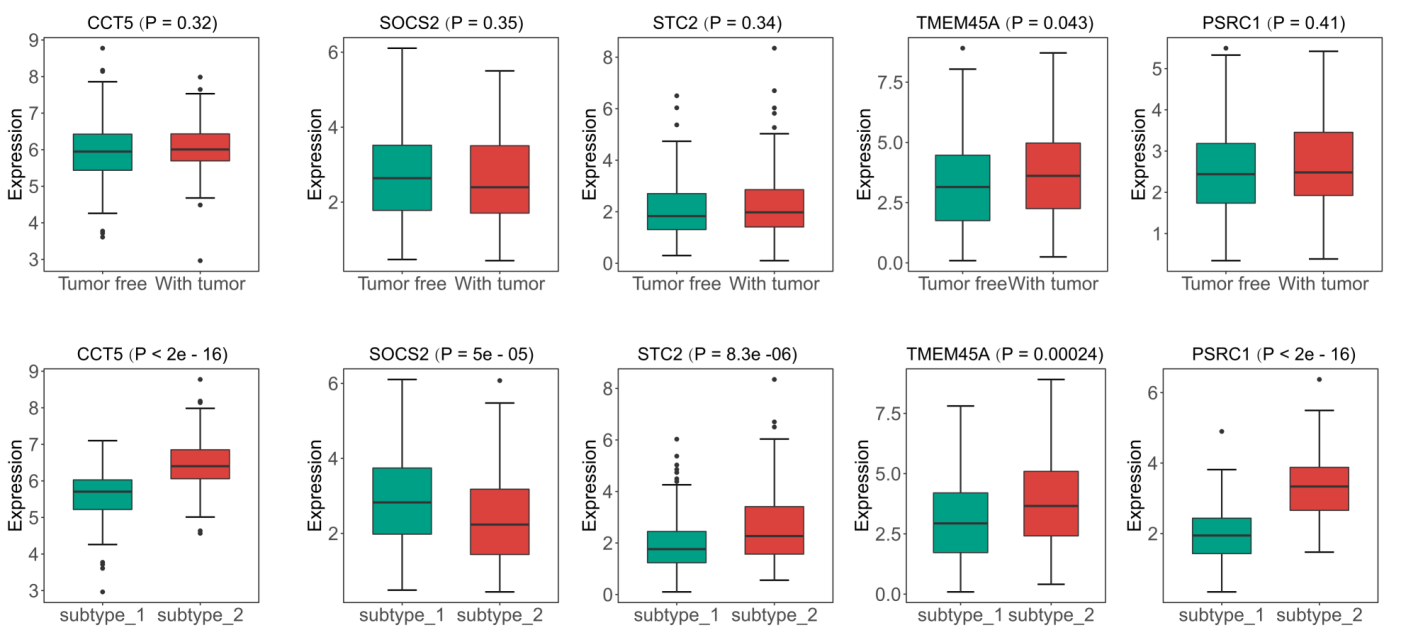


**Supplementary Figure 7 Comparison of the expression of the five CSGscore genes between subsets of tumor burden and CSG subtype.**
